# *Potentilla tormentilla* Extract Loaded Gel: Formulation, *In Vivo* and *In Silico* Evaluation of Anti-Inflammatory Properties

**DOI:** 10.3390/ijms25179389

**Published:** 2024-08-29

**Authors:** Jovana Bradic, Anica Petrovic, Milos Nikolic, Nikola Nedeljkovic, Marijana Andjic, Jovan Baljak, Vladimir Jakovljevic, Aleksandar Kocovic, Vanja Tadic, Aleksandra Stojanovic, Igor Simanic

**Affiliations:** 1 Department of Pharmacy, Faculty of Medical Sciences, University of Kragujevac, 34000 Kragujevac, Serbia; jovanabradickg@gmail.com (J.B.); petkovicanica0@gmail.com (A.P.); nikola.nedeljkovic@fmn.kg.ac.rs (N.N.); andjicmarijana10@gmail.com (M.A.); salekkg91@gmail.com (A.K.); vranicaleksandra90@gmail.com (A.S.); 2 Center of Excellence for Redox Balance Research in Cardiovascular and Metabolic Disorders, 34000 Kragujevac, Serbia; drvladakgbg@yahoo.com; 3 Department of Pharmacy, Faculty of Medicine, University of Novi Sad, 21000 Novi Sad, Serbia; baljakjovan99@yahoo.com; 4 Department of Physiology, Faculty of Medical Sciences, University of Kragujevac, 34000 Kragujevac, Serbia; 5 Department of Human Pathology, 1st Moscow State Medical University IM Sechenov, 119991 Moscow, Russia; 6 Institute for Medicinal Plant Research “Dr. Josif Pančić”, 11000 Belgrade, Serbia; vtadic@mocbilja.rs; 7 Specialized Hospital for Rehabilitation and Orthopedic Prosthetics, Sokobanjska 17, 11000 Beograd, Serbia; dr.igorsimanic@yahoo.com; 8 Department of Physical Medicine and Rehabilitation, Faculty of Medical Sciences, University of Kragujevac, 69 Svetozara Markovica St., 34000 Kragujevac, Serbia

**Keywords:** *Potentilla tormentilla*, gel, anti-inflammatory activity, *in silico* studies, rats

## Abstract

The objective of the study was to develop a novel topical gel by mixing *Potentilla tormentilla* ethanolic extract, thermosensitive poloxamer 407, and carbomer 940 and evaluating its stability and rheological behavior. The irritation potential of the gel was evaluated in accordance with the Organization for Economic Cooperation and Development Guidelines 404. The potential anti-inflammatory effects of the developed gel were evaluated *in vivo* in rats using the carrageenan-induced paw edema test. Moreover, the *in silico* binding affinity for chlorogenic and ellagic acid, as dominant components in the extract, against cyclooxygenase (COX) 1 and 2 was also determined. Our findings suggest that the gel containing *Potentilla tormentilla* extract remained stable throughout the observation period, exhibited pseudoplastic behavior, and caused no irritation in rats, thus being considered safe for topical treatment. Additionally, the developed gel showed the capability to reduce rat paw edema, which highlights significant anti-inflammatory potential. *In silico* analysis revealed that chlorogenic and ellagic acid exhibited a reduced binding affinity against COX-1 but had a similar inhibitory effect on COX-2 as flurbiprofen, which was confirmed by molecular dynamics results. The study proposes the possible application of *Potentilla tormentilla* ethanolic extract gel for the alleviation of localized inflammatory diseases; however, future clinical evaluation is required.

## 1. Introduction

In recent years, more attention in the cosmetic and pharmaceutical industries has been dedicated to plants and plant extracts that are considered an alternative approach used to mitigate and prevent the development of different health and skin problems. Natural ingredients have been used for centuries for skin treatment and care, mostly due to the fact that they are easily absorbed and metabolized by the human body but also show fewer side effects than conventional medications [1]. There are numerous traditional plants for which there is considerable interest in their use for dermatological products due to their valuable phytochemical potential [2].

The *Potentilla* genus has been known since ancient times for its curative properties, traditionally for skin injuries such as wounds and burns [3,4]. *Potentilla tormentilla* (synonyms: *Potentilla erecta*, *Tormentilla erecta*), as one of the most prominent species of the *Potentilla* genus, has mainly spread in the temperate, Arctic, and alpine zones of the northern hemisphere [3,4]. This plant species has been traditionally used due to its anti-inflammatory, antioxidative, anti-ulcerogenic, and antimicrobial curative properties [5]. The therapeutic properties of *Potentilla tormentilla* mostly come from its rhizome, rich in active pharmacological compounds such as tannins, astringent polyphenols (up to 20%), and especially the hydrolysable tannin agrimoniin [6]. Agrimoniin has an outstanding capacity as an antioxidant, astringent, and elastase inhibitor comparable to the most potent anti-allergic drug, azelastine, and about 20 times more effective than epigallocatechin gallate, the main tannin of green tea [3]. 

Skin inflammation has been associated with numerous dermal pathological conditions such as psoriasis, atopic dermatitis, eczema, and seborrheic dermatitis [7]. Therefore, great effort has been invested in the development of naturally based topical formulations that can exert anti-inflammatory potential and improve the management of patients with skin diseases. *Potentilla tormentilla* stands out as a source of valuable compounds with anti-inflammatory properties that can be used as ingredients for the development of potent dermocosmetic products. In addition to traditional usage, previous research confirmed that bioactive compounds present in *Potentilla tormentilla* extract can reduce UVB-induced inflammation in *in vitro* and *in vivo* models [6]. The anti-inflammatory effect of *Potentilla tormentilla* is based on lowering the formation of interleukin-6 and prostaglandin E2 as principal mediators of inflammation. Importantly, it has been reported that this plant species shows an effect comparable to hydrocortisone, thus paving the way for using *Potentilla tormentilla* extract as an active component in developing drug delivery systems for the treatment of inflammatory skin disorders [8]. 

Previous studies predominantly highlight the *in vitro* anti-inflammatory properties of *Potentilla tormentilla* extract [4,9]. However, there is a notable lack of scientific data addressing the effects of local application of *Potentilla tormentilla* extract, whether used alone or incorporated into specific delivery vehicles. This gap underscores the need for further research to explore the efficacy and practical applications of *Potentilla tormentilla* extract in topical formulations. Therefore, our study introduces innovation through a specific formulation that utilizes *Potentilla tormentilla* extract in combination with novel polymeric bases, filling existing gaps and providing new insights into its application. 

The significance of selecting the right polymer for creating topical gels lies in its impact on the gel’s overall performance and user experience. With a vast array of polymers available, choosing the appropriate one ensures optimal gel consistency, stability, and controlled release of active ingredients. This careful selection affects how well the gel adheres to the skin, how effectively it delivers the therapeutic agents, and how comfortable it is for the user, ultimately influencing the treatment’s efficacy and patient compliance.

Regarding the above-mentioned data, our study aimed, for the first time, to develop a novel topical gel formulation using *Potentilla tormentilla* extract combined with poloxamer 407 and carbomer 940 for anti-inflammatory activity. This combination could potentially allow for more effective delivery of bioactive compounds and thus enhance the anti-inflammatory effect of *Potentilla tormentilla* extract. Accordingly, the special novelty of the current research is reflected in the development of an efficient delivery system for *Potentilla tormentilla* extract that increases the extract’s efficacy through prolonged maintenance on the skin, as well as providing an in-depth understanding of its anti-inflammatory potential in an animal model.

## 2. Results

### 2.1. Chemical Composition of Potentilla Tormentilla Extract

The results of preliminary chemical characterization indicated that the TPC of *Potentilla tormentilla* ethanolic extract was 328.4 ± 24.74 mg GA/d.w. extract, whereas the TTC was 3.54 ± 0.13% (expressed as pyrogallol, *w*/*w*).

The chemical composition of the *Potentilla tormentilla* ethanolic extract is shown in Table 1 and Figure S1.

The results indicate that the most abundant single phenolic compound was ellagic acid, followed by somewhat lower amounts of epicatechin. Furthermore, significantly less abundance of gallic and chlorogenic acid was recorded, accompanied by traces of the flavonoid heteroside rutin. However, we must add to the aforementioned that the applied instrumental technique enabled us to non-specifically identify and quantitate high amounts of different ellagic acid derivatives present in the analyzed extract.

### 2.2. Physicochemical Characterization of PEG

#### 2.2.1. Organoleptic Characteristics and Physical Appearance

Parameters of formulated gel such as colour, odour, consistency, and homogeneity, observed at different temperatures during three months’ storage, are presented in Table 2. The results indicated that the organoleptic characteristics and homogeneity of the prepared formulation remained unaffected at different temperatures during the three months. 

#### 2.2.2. pH Values

The results of pH value measurements are presented in Table 3. Our gel formulations did not change in terms of pH during storage period, thus suggesting that it does not have the potential to disturb the skin barrier, but that it supports natural skin pH balance. Importantly, formulations stored at both temperatures had pH values that are suitable and recommended for topical products [10].

#### 2.2.3. Electrical Conductivity

Another important parameter for water-based products is electrical conductivity. In this investigation, we focused on electrical conductivity values during the storage time at different temperatures, which is presented in Table 4. Our results showed that storing PEG on 25 °C led to a slight decrease in conductivity, while storing PEG at a lower temperature led to significant elevation of conductivity values.

#### 2.2.4. Centrifugation Test of Prepared Formulation

After centrifugation of PEG, there was no noticeable decomposition, separation, or precipitation of the components of the gel formulation. This proved to us the stability of the gel formulation.

#### 2.2.5. Rheological Characterization of Prepared Formulation

**Figure 1:**
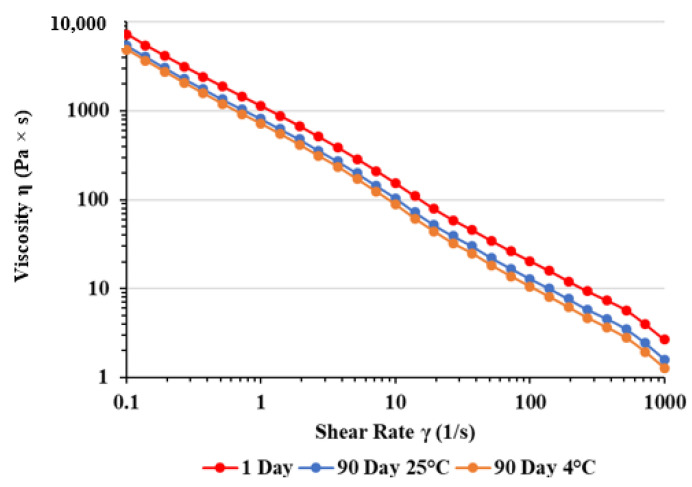
Viscosity curves of PGE after preparation and three months of storage at different temperatures measured at 25 °C. For curve 1 day: y = 1074.2 × x −0.85, R^2^ = 0.9992; for curve 90 day 25 °C: y = 766.6 × x −0.87, R^2^ = 0.9992; for curve 90 day 4 °C: y = 673.9 × x −0.89, R^2^ = 0.9992.

**Figure 2:**
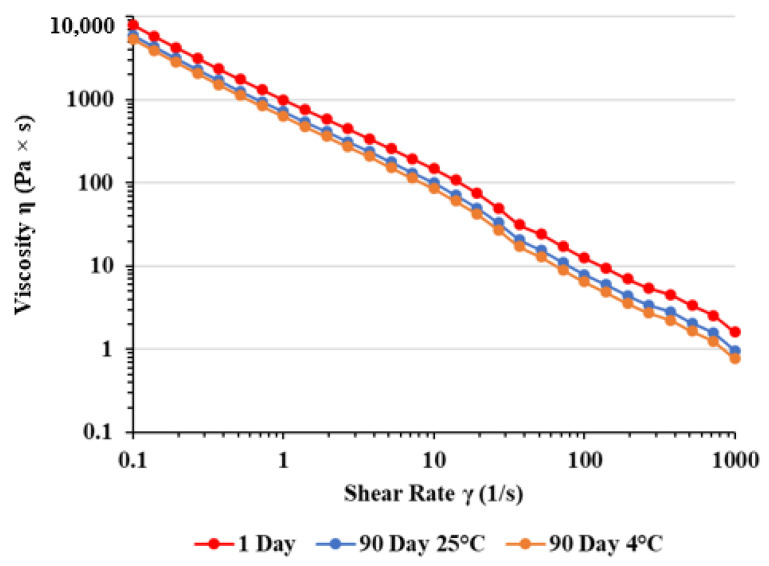
Viscosity curves of PGE after preparation and three months of storage at different temperatures measured at 37 °C. For curve 1 day: y = 1014.3 × x −0.92, R^2^ = 0.9983; for curve 90 day 25 °C: y = 723.8 × x −0.95, R^2^ = 0.9983; for curve 90 day 4 °C: y = 636.3 × x −0.96, R^2^ = 0.9982.

Figure 1 and Figure 2 represent the viscosity curves, which describe the dependence of the viscosity of PEG on shear rates at temperature of 25 °C and 37 °C (Table 5 and Table 6).

The results shown in Figure 1 and Figure 2 demonstrate that the PEG showed shear thinning behavior with viscosity decreasing as the shear rate was increased.

#### 2.2.6. Swelling Index of Prepared Formulation

The swelling index of the PGE formulation on the first day and after 90 days of storage at 4 ± 2 °C and 25 ± 2 °C is represented in Table 7. The gel formulation with PGE showed a higher swelling index in the third hour of measurement in comparison to the zero and first hour after the preparation of the sample. The highest swelling index was noticed in the PGE gel after three months of storage at room temperature. 

### 2.3. Acute Dermal Irritation Test

**Figure 3:**
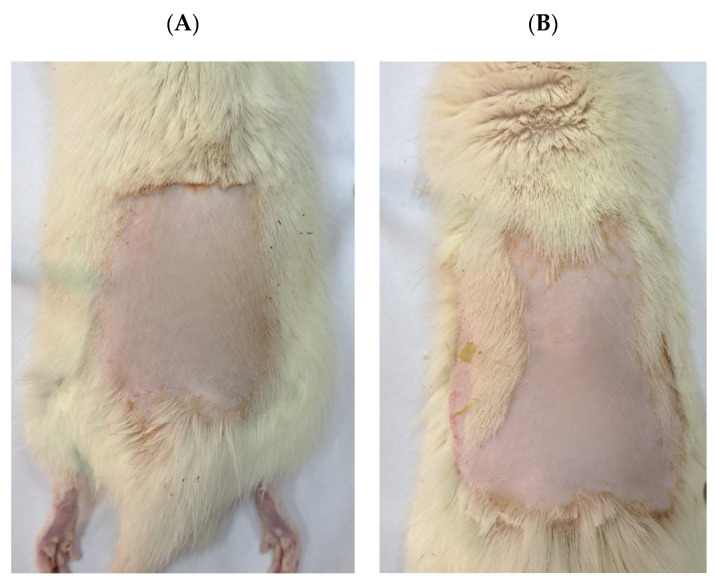
Rat skin after application of BG (**A**) and PEG (**B**).

The animals subjected to topical application of PEG showed no signs of acute dermal toxicity such as erythema, edema, and other clinical toxicity manifestations during 14 days of observation (Figure 3). In brief, the absence of other clinical toxicity manifestations includes neither skin effects (e.g., defatting of skin) nor any systemic adverse effects such as reduction in body weight, any alterations in behavior patterns, urination (colour) and faeces consistency alterations, salivation alterations, etc.

### 2.4. Anti-Inflammatory Activity of PEG in Rat Model

**Figure 4:**
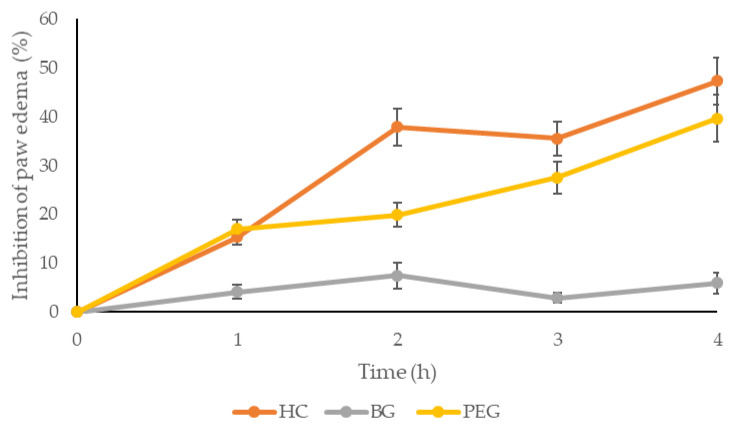
Percentage of paw edema inhibition in rats treated with formulations. HC—hydrocortisone; BG—gel base; PEG—*Potentilla* extract-based gel.

The results of the *in vivo* anti-inflammatory activity of PEG are shown in Table 8 and Figure 4. There was no difference between the examined groups one hour after carrageenan injection. Nevertheless, the administration of PEG led to significant inhibition of paw edema after the second, third, and fourth hour of carrageenan application compared to the control group. The application of a gel base did not decrease inflammation, which was expected due to the absence of therapeutic active compounds. Hydrocortisone as a standard anti-inflammatory drug showed the most pronounced degree of inhibition compared to the control group.

### 2.5. Molecular Docking Simulations

**Figure 5:**
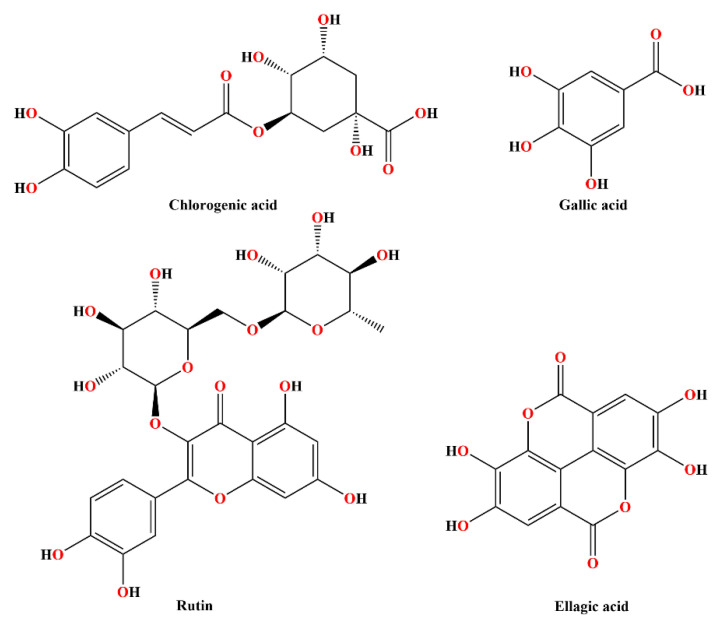
Chemical structures of tested polyphenolic compounds.

Considering the prominent anti-inflammatory activity exhibited by the investigated extracts, molecular docking studies were used to evaluate the binding affinity of four polyphenolic compounds present in the extracts (Figure 5) towards COX-1 and COX-2 isozymes.

**Figure 6:**
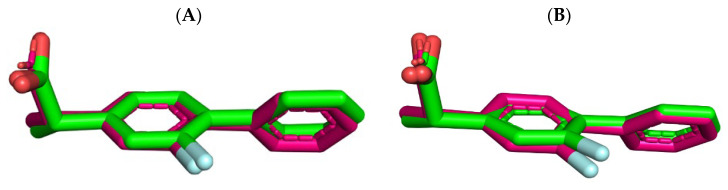
The superimposition of the co-crystallized flurbiprofen (green) and re-docked binding conformation of flurbiprofen (hot pink) within the active site of COX-1 (**A**) and COX-2 (**B**).

To validate the docking protocol, the co-crystallized ligand flurbiprofen was extracted and subsequently re-docked within the binding sites of the target enzymes. The computed Root Mean Square Deviation (RMSD) values of 0.3007 Å for COX-1 and 0.2851 Å for COX-2 indicated the reliability of the conducted docking procedure (Figure 6A,B).

The binding attributes for the most favorable docking poses of the investigated compounds are delineated in Table 9. A lower estimated value of docking score (ΔG) and inhibition constant (K_i_) signify a more potent molecular interaction between the investigated compound and protein. The docking scores ranged from −28.45 to −10.88 kJ/mol for COX-1 and from −34.72 to −25.94 kJ/mol for COX-2, among the tested compounds. 

Correspondingly, the calculated inhibition constants ranged from 10.20 to 12,300 µM for COX-1 and from 0.808 to 28.09 µM for COX-2. Among the tested compounds, chlorogenic acid and ellagic acid stand out as the molecules displaying the highest binding affinity, as evident from the lowest values of docking score and inhibition constant. These values, though higher, are comparable to those observed for flurbiprofen, as opposed to gallic acid and rutin, which exhibited significant lower binding affinity in comparison to flurbiprofen.

**Figure 7:**
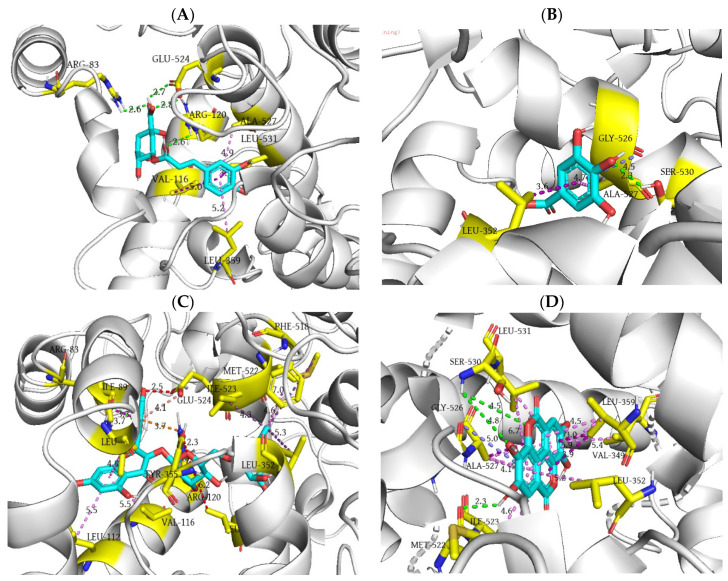
Three-dimensional representation of chlorogenic acid (**A**), gallic acid (**B**), rutin (**C**), and ellagic acid (**D**) binding interactions within the active site of COX-1. The depicted interactions along with their corresponding bond lengths (Å) include conventional hydrogen bonds (green dashed lines), C–H hydrogen bonds (pale green dashed lines), alkyl interactions (violet purple dashed lines), π-σ interactions (purple dashed lines), π-π interactions (hot pink dashed lines), π–alkyl interactions (violet dashed lines), amide–π interactions (slate blue dashed lines), π–cation interactions (orange dashed lines), π–anion interactions (red salmon dashed lines), and unfavorable steric bumps (red dashed lines).

Based on the results presented in Table 9, it can be concluded that chlorogenic acid shows the highest binding affinity towards the COX-1 isoform. Namely, this compound achieves the lowest values of docking score (−28.45 kJ/mol) and inhibition constant (10.20 µM). It may be hypothesised that the superior affinity of chlorogenic acid towards COX-1, compared to other investigated compounds, is due to the formation of four hydrogen bonds. Specifically, arginine and glutamic acid at positions 83 and 524, respectively, form a single hydrogen bond, while arginine at position 120 is double hydrogen-bonded in its interaction with the mentioned ligand. The carbonyl oxygen atom within the ester group of chlorogenic acid interacts as a hydrogen bond acceptor with the guanidine moiety of the ARG120 residue. The identical group of the specified residue establishes a contact with the hydroxyl oxygen atom within the carboxyl group of chlorogenic acid, acting as a hydrogen bond donor. The guanidine group of the ARG83 residue, as a proton donor, establishes a hydrogen bond with the carbonyl oxygen atom within the carboxyl group of the investigated ligand. Conversely, the only hydrogen bond where the ligand acts as a proton donor is the contact established between the hydroxyl hydrogen atom of chlorogenic acid’s carboxyl group and the carboxyl oxygen atom of the GLU524 residue. Other interactions that contribute to the stability of the formed complex include hydrophobic contacts of the π-σ type between the ligand’s aromatic ring and residues VAL116 and LEU531, as well as π–alkyl interactions involving the same region of chlorogenic acid and residues LEU359 and ALA527 (Figure 7A). 

A slightly higher value of the docking score of gallic acid compared to chlorogenic acid can be explained by the formation of only one hydrogen bond with the dominant presence of hydrophobic contacts. Concretely, the oxygen atom of the hydroxyl group in position 3 of gallic acid acts as a hydrogen bond acceptor, establishing a contact with the hydroxyl hydrogen atom of the residue SER530. The remaining hydrophobic interactions of π-σ, amide–π stacked, and π–alkyl types are formed between the aromatic ring and residues LEU352, GLY526, and ALA527, respectively (Figure 7B). 

Upon the molecular docking of rutin into the active site of COX-1, it was observed that this molecule exhibits the lowest affinity towards the target enzyme. Namely, the highest values of free binding energy (−10.88 kJ/mol) and inhibition constant (12,300 µM) were calculated, regardless of the presence of the double hydrogen bond formed by the mentioned compound and residue SER530. The low binding affinity of rutin may be attributed to the presence of three unfavorable interactions that significantly hinder the molecular fitting of this compound, in addition to numerous hydrophobic interactions. More precisely, the pyranic oxygen atom of the 6-hydroxymethyl-tetrahydro-pyran-2,3,4,5-tetraol group and the phenolic oxygen atom of the 3,4-dihydroxyphenyl group of rutin form two unfavorable acceptor–acceptor interactions with residues TYR355 and GLU524, respectively. On the other hand, the hydroxyl oxygen atom in position 3 of the 6-hydroxymethyl-tetrahydro-pyran-2,3,4,5-tetraol nucleus establishes an unfavorable bump with the alkyl group within the side chain of the LEU531 residue (Figure 7C).

Based on the values of free binding energy, it can be concluded that ellagic acid (−26.36 kJ/mol) exhibits binding affinity towards COX-1 subsequent to chlorogenic acid. The relatively high affinity of this molecule against the target enzyme can be explained by the formation of three hydrogen bonds. Namely, the phenolic group of ellagic acid forms a single hydrogen bond as an H-donor with the carbonyl oxygen of MET522. On the other hand, the hydroxyl group of SER530 is double hydrogen-bonded as a proton donor with the phenolic and lactone oxygen atoms of the ligand. The establishment of a significant number of hydrophobic interactions (14 interactions) further contributes to the relatively high stability of the formed ligand–protein complex. Among them, the most numerous are the π–alkyl interactions, wherein the π electrons of the tetracyclic core of ellagic acid interact with the side chains of LEU352, LEU359, LEU523, and LEU531 residues. Other types of hydrophobic interactions include amide–π (GLY526) and π-σ contacts (VAL349 and ALA527) (Figure 7D).

**Figure 8:**
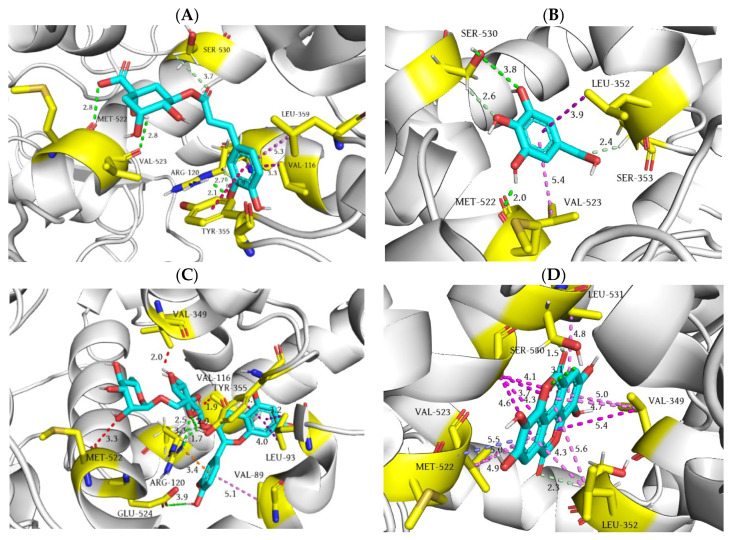
Three-dimensional representation of chlorogenic acid (**A**), gallic acid (**B**), rutin (**C**), and ellagic acid (**D**) binding interactions within the active site of COX-2. The depicted interactions along with their corresponding bond lengths (Å) include conventional hydrogen bonds (green dashed lines), C-H hydrogen bonds (pale green dashed lines), π-σ interactions (purple dashed lines), π-π interactions (hot pink dashed lines), π–alkyl interactions (violet dashed lines), amide–π interactions (slate blue dashed lines), π–cation interactions (orange dashed lines), and unfavorable steric bumps (red dashed lines).

During molecular docking of selected polyphenols into the binding site of COX-2, it is evident that chlorogenic acid demonstrates the lowest docking score value (−34.72 kJ/mol), as well as the lowest inhibition constant (0.808 µM). These results suggest that chlorogenic acid exhibits a comparable binding affinity to the conventional NSAID flurbiprofen (−38.07 kJ/mol and 0.209 µM). Namely, one of the phenolic groups of chlorogenic acid establishes an identical hydrogen bond with residue ARG120 as flurbiprofen, with the difference that the carboxyl group of the drug is double hydrogen-bonded with the mentioned residue. Moreover, the hydroxyl oxygen atom of chlorogenic acid’s carboxyl group and adjacent secondary hydroxyl group act as proton donors in the hydrogen bond interaction with carbonyl oxygen atoms of MET522 and VAL523 peptide bonds. Furthermore, the side chains of residues ARG120 and SER530 are involved in the formation of two additional C-H hydrogen bonds with the aforementioned secondary amino group of chlorogenic acid, which is engaged in the formation of a conventional hydrogen bond with guanidine moiety of ARG120, as well as with the carbonyl oxygen of the ester functional group. The notable binding affinity of chlorogenic acid towards COX-2 can be additionally attributed to the formation of three hydrophobic interactions, wherein the phenyl ring of the molecule establishes π-σ, π-π, and π–alkyl contacts with residues VAL116, TYR355, and LEU359, respectively (Figure 8A).

Molecular docking of gallic acid into the binding site of COX-2 reveals that two hydroxyl groups in positions 3 and 5 are involved in the formation of two conventional hydrogen bonds with residues MET522 and SER530, wherein 3-OH acts as a hydrogen bond donor (bond length 2.0 Å), while the 5-OH group participates as a hydrogen bond acceptor (bond length 3.8 Å). In addition, the carbon atoms of the two serine residues in positions 353 and 530 interact with the carbonyl oxygen atom of the carboxyl group and 4-OH group of gallic acid, establishing two C-H hydrogen bonds. Eventually, the carbon atoms of the isobutyl group (LEU352) and isopropyl group (VAL523) form π-σ and π–alkyl hydrophobic interactions with the phenyl core of gallic acid (Figure 8B).

Despite the fact that the oxygen atoms of the 6-hydroxymethyl-tetrahydro-pyran-2,3,4,5-tetraol moiety establish multiple hydrogen bonds with residues ARG120 and TYR355, similar to flurbiprofen, and the 4H-chromen-4-one core interacts with residues VAL89, LEU93 and VAL116, establishing four hydrophobic interactions in the binding site of COX-2, rutin accomplishes relatively high values of docking score (−27.61 kJ/mol) and inhibition constant (14.30 µM) compared to flurbiprofen (−38.07 kJ/mol and 0.209 µM). The explanation for the substantial difference in binding affinity between these two compounds can be attributed to the occurrence of three unfavorable interactions encountered during the molecular docking of rutin into the binding site of COX-2. Specifically, the isopropyl side chains of residues VAL116 and VAL349 form two unfavorable steric bumps with the hydroxyl groups of the tetrahydrofuran cores, whereas the carbonyl oxygen atom of the MET522 peptide bond establishes an unfavorable acceptor–acceptor interaction (Figure 8C).

In the molecular interaction of ellagic acid and the active site of COX-2, it is evident that the investigated molecule exhibits a very similar docking score (−34.22 kJ/mol) as chlorogenic acid (−34.72 kJ/mol), and thus a comparable binding affinity with respect to flurbiprofen (−38.07 kJ/mol). The relative high affinity of ellagic acid towards COX-2 is attributed dominantly to the formation of multiple hydrophobic interactions (13 in total) between its tetracyclic core and amino acids within COX-2’s hydrophobic pocket. Specifically, the hydrophobic side chains of residues VAL349, LEU352, VAL523, ALA527, and LEU531 establish a grid of hydrophobic π–alkyl interactions with the π electronic clouds of ellagic acid’s aromatic rings. Interestingly, residue ALA527 forms hydrophobic interactions with all four aromatic rings of ellagic acid. Moreover, the peptide bond between residues MET522 and VAL523 and the adjacent aromatic ring establish an additional hydrophobic amide–π interaction. Finally, intramolecular lactones of ellagic acid contribute to the stabilization of the ellagic acid–COX-2 complex through the formation of a single conventional (SER530) and two C-H hydrogen bonds with the side chains of residues SER530 and SER353 (Figure 8D).

The data presented in Table 9 regarding the LE values demonstrate that this parameter does not significantly determine the docking score value, as evidenced by minimal differences in the binding energy per ligand atom obtained for tested compounds. The LE values ranged from −2.13 to −0.25 kJ/mol for COX-1 and from −2.16 to −0.64 kJ/mol for COX-2, with the lowest values observed for gallic acid during its molecular fitting into the active sites of both enzymes. This observation is unsurprising, given that gallic acid has the smallest molecular size among the investigated molecules. 

### 2.6. Molecular Dynamics Simulations and MM/GBSA Computation

Chlorogenic acid and ellagic acid were selected for MD analyses according to their high binding affinity towards COX-1 and COX-2, established in molecular docking studies. Accordingly, the docking poses of chlorogenic acid and ellagic acid in the binding sites of target enzymes were used as initial binding modes in MD simulations. Within the MD analyses, the conformational stability of chlorogenic acid–COX-1, ellagic acid–COX-1, chlorogenic acid–COX-2, and ellagic acid–COX-2 complexes over time was examined, as well as flurbiprofen complexes with COX-1 and COX-2, which served as conformational controls.

**Figure 9:**
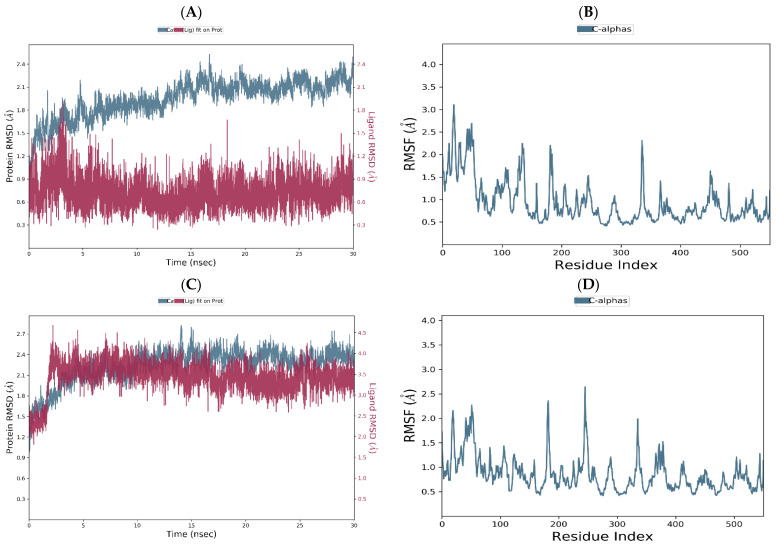

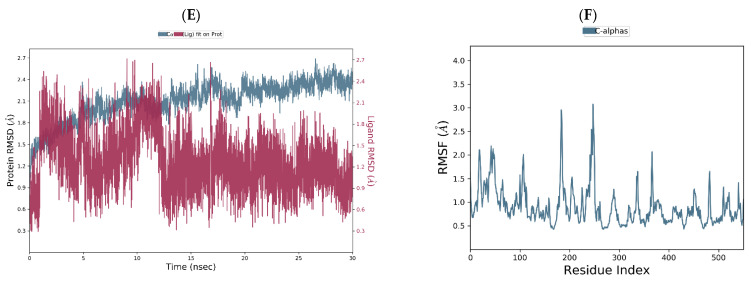
RMSD of the Cα of COX-1 and flurbiprofen plotted versus simulation time (**A**). The fluctuations in RMSF of COX-1 Cα with respect to the residue index observed for the flurbiprofen–COX-1 complex (**B**). RMSD of the Cα of COX-1 and chlorogenic acid plotted versus simulation time (**C**). The fluctuations in RMSF of COX-1 Cα with relation to the residue index observed for the chlorogenic acid–COX-1 complex (**D**). RMSD of the Cα of COX-1 and ellagic acid plotted versus simulation time (**E**). The fluctuations in RMSF of COX-1 Cα with relation to the residue index observed for the ellagic acid–COX-1 complex (**F**).

The RMSD plot of the flurbiprofen–COX-1 complex displays the moderate deviations in RMSD values of both ligand (in order 0.8–1.3 Å) and protein (in order 1.2–5.3 Å), indicating their conformational stability across the simulation (Figure 9A). The corresponding RMSF plot demonstrates minor local fluctuations along the protein chain, with values slightly above 3 angstroms (Figure 9B).

The RMSD diagram of the chlorogenic acid–COX-1 complex shows the conformational stability throughout the entire duration of the simulation. Certain deviations were observed between 3 and 4 ns of the simulation, which is not particularly significant in relation to the total duration of the simulation process. Thereupon, the complex stabilized until the end of the simulation. Protein deviations were present in the interval from 2 to 4.5 Å, while values of the ligand ranged from 1 to 2.8 Å (Figure 9C). The RMSF plot shows small local deviations along the protein chain, with the exception of residues GLY278 and LYS215 (values of about 2 Å), which are not significant for ligand binding to the target enzyme (Figure 9D).

The ellagic acid–COX-1 complex achieves a higher level of conformational stability compared to the co-crystal complex with the same target enzyme, but less overlap compared to the chlorogenic acid–COX-1 complex. As depicted in Figure 9E, the RMSD values of the protein increase in the first 5 ns, followed by stabilization with the presence of fluctuations in the interval from 1.8 to 2 Å. In contrast, the RMSD plot of ellagic acid indicates the presence of conformational instability observed during the initial 12 ns of the simulation, after which conformational stabilization occurs with the presence of minimal absolute value of fluctuations around 1.5 Å. The RMSF diagram shows two pronounced deviations of the MET216 and PRO280 residues, exhibiting RMSF values of 2.96 and 3.08 Å, respectively (Figure 9F). However, these residues are not important during the molecular fitting of ellagic acid into the COX-1 binding site.

**Figure 10:**
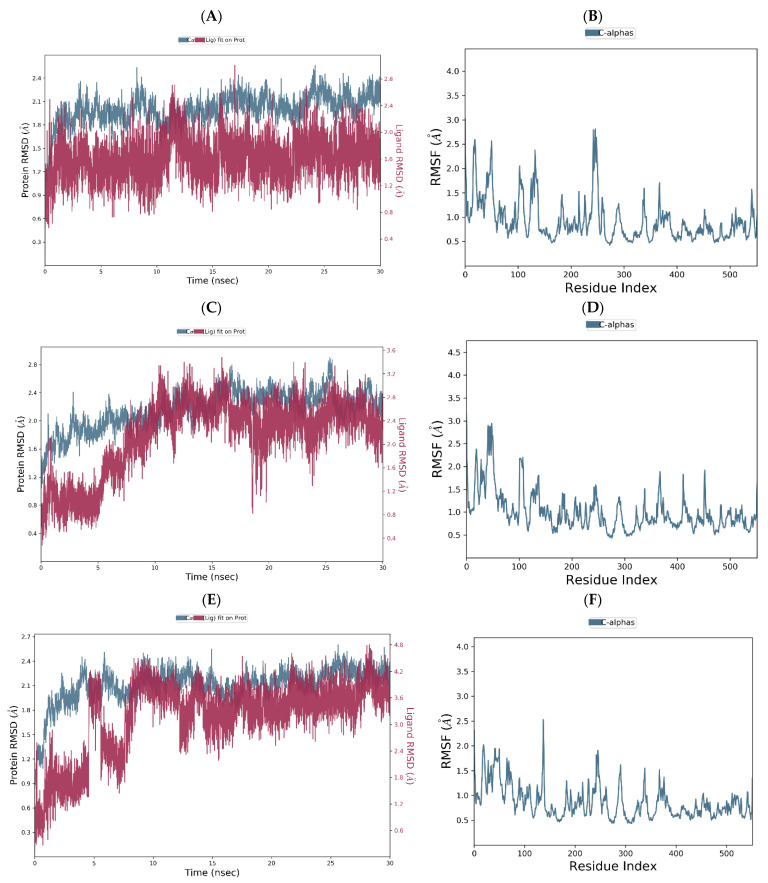
RMSD of the Cα of COX-2 and flurbiprofen plotted versus simulation time (**A**). The fluctuations in RMSF of COX-2 Cα with respect to the residue index observed for the flurbiprofen–COX-2 complex (**B**). RMSD of the Cα of COX-2 and chlorogenic plotted versus simulation time (**C**). The fluctuations in RMSF of COX-2 Cα with relation to the residue index observed for the chlorogenic acid–COX-2 complex (**D**). RMSD of the Cα of COX-2 and ellagic acid plotted versus simulation time (**E**). The fluctuations in RMSF of COX-2 Cα with relation to the residue index observed for the ellagic acid–COX-2 complex (**F**).

The flurbiprofen–COX-2 RMSD plot demonstrates consistent conformational stability across the entire simulation, as indicated by the overlap of ligand and protein deviations (Figure 10A). As a consequence of the interaction between flurbiprofen and COX-2, minor local alterations were observed for isoleucine and histidine residues at positions 274 and 278, respectively, with RMSF values below 3 angstroms (Figure 10B). Analogously to the flurbiprofen–COX-2 complex, the RMDS plots of chlorogenic acid and COX-2 are mutually overlapped in the last 20 ns of the simulation, suggesting the retention of chlorogenic acid within its initial binding site and the formation of a relatively stable complex during the simulation period. Although the moderate deviations of the order 1.2–2.8 Å can be observed in the protein RMSD diagram, these values indicate that the protein does not undergo substantial conformational changes during the simulation (Figure 10C). The chlorogenic acid–COX-2 RMSF diagram revealed slight local changes for residues PHE74 (2.90 Å), ILE78 (2.88 Å), and LEU81 (2.94 Å); however, these residues are not directly involved in ligand binding within the binding site of COX-2. Eventually, the observed conformational stability of residues involved in the molecular docking of chlorogenic acid into the active site of COX-2 (VAL116, ARG120, TYR355, LEU359, MET522, VAL523, and SER530) suggests a similar binding mode of flurbiprofen and chlorogenic acid (Figure 10D).

Similar to the chlorogenic acid–COX-2 complex, the RMSD diagram of ellagic acid–COX-2 complex illustrates the overlapping of the ligand and protein charts, indicating system equilibration and the formation of a stable ligand–protein complex in the last 20 ns of the simulation. However, in contrast to the chlorogenic acid–COX-2 complex, significant protein RMSD deviations in the ellagic acid–COX-2 complex are not observed in this simulation period, suggesting that the protein maintains its conformational stability during the simulation (Figure 10E). The ellagic acid–COX-2 RMSF diagram shows only one dominant deviation for residue LYS169 (2.53 Å), which does not directly participate in the molecular docking of the ligand into the binding site of COX-2 (Figure 10F).

**Figure 11:**
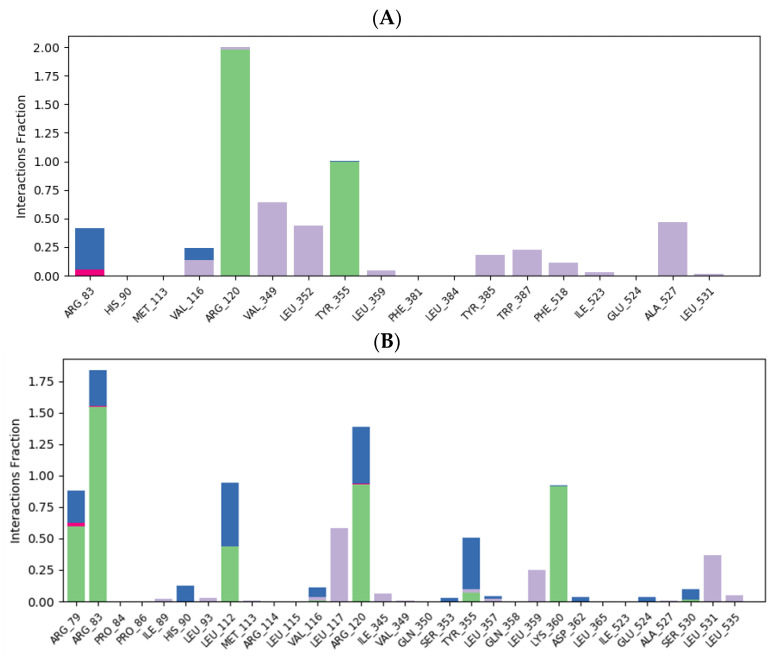

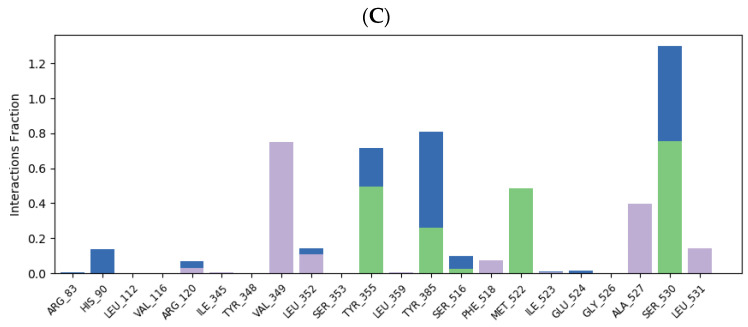
Flurbiprofen–COX-1 (**A**), chlorogenic acid–COX-1 (**B**), and ellagic acid–COX-1 (**C**) ligand–protein plots of binding interactions during MD simulation. Green bars (hydrogen bonds), red bars (ionic interactions), grey bars (hydrophobic interactions), and blue bars (water bridges) are depicted.

**Figure 12:**
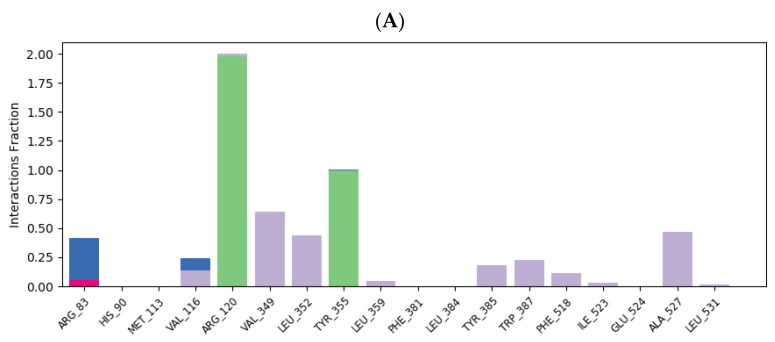

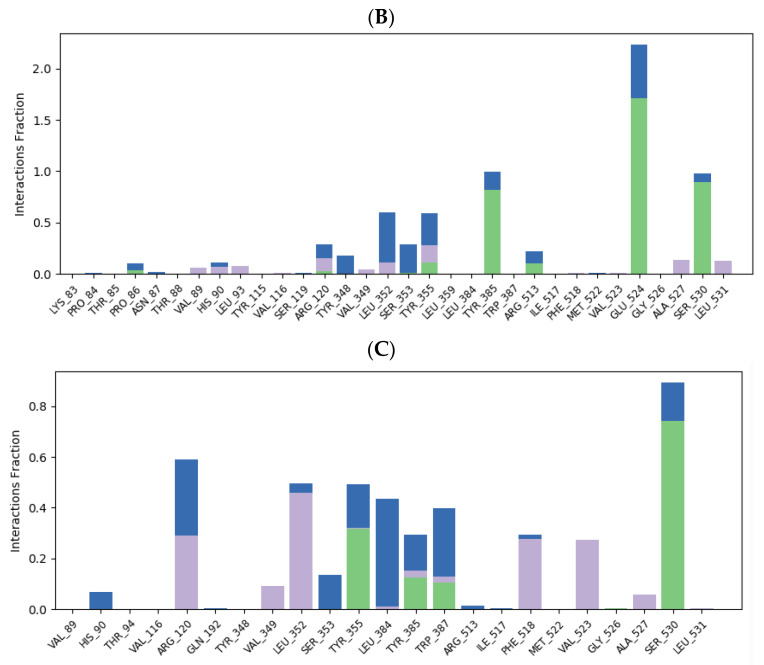
Flurbiprofen–COX-2 (**A**), chlorogenic acid–COX-2 (**B**), and ellagic acid–COX-2 (**C**) ligand–protein plots of binding interactions during MD simulation. Green bars (hydrogen bonds), red bars (ionic interactions), grey bars (hydrophobic interactions), and blue bars (water bridges) are depicted.

The examination of ligand–protein contacts was carried out to identify the binding contacts present for the most of the simulation time. Figure 11 and Figure 12 illustrate the distribution of binding interaction fractions involved in the stabilization of the following complexes: flurbiprofen–COX-1, chlorogenic acid–COX-1, ellagic acid–COX-1, flurbiprofen–COX-2, chlorogenic acid–COX-2, and ellagic acid–COX-2, during MD simulations. According to the ligand–protein interaction diagram, it can be observed that flurbiprofen, as a co-crystallized ligand, establishes two dominant hydrogen bond interactions with residues ARG120 and TYR355, persisting for nearly 200% and 100% of the simulation duration, respectively. The stability of the formed complex is additionally contributed by hydrophobic contacts with amino acids VAL349, LEU352, and ALA527, which are maintained approximately 75%, 40%, and 50% of the time, respectively (Figure 11A). Arginine residues at positions 83 and 120 predominantly contribute to the stabilization of the chlorogenic acid–COX-1 complex, primarily through the establishment of multiple hydrogen bonds. Examination of ligand-protein contacts revealed that these interactions are present during MD simulation with time fractions of 154.9% and 93.2%, respectively. It can be noticed that the hydrophobic interaction that most contributes to the stability of the chlorogenic acid–COX-1 complex is the contact of the ligand’s aromatic ring with the LEU531 residue that is represented during 36.7% of the simulation period (Figure 11B). When it comes to the complex of ellagic acid with COX-1, we can conclude that the interaction with the residue SER530 is the most frequent; 75.7% of the simulation time, it has the character of a hydrogen bond, while 54.4% of the simulation duration, it represents a water bridge. The only remaining hydrogen interaction significant for ellagic acid binding to COX-1 (with the MET522 residue) is present 48.7% of the time. The other hydrogen bond and water bridge contacts (TYR355 and TYR385), present during almost the entire duration of the simulation, are not identified using the molecular docking study. Concerning hydrophobic interactions, contacts with residues VAL349 (74.9%) and ALA527 (39.8%) contribute the longest to the stability of the ellagic acid–COX-1 complex (Figure 11C).

The ligand–protein interaction diagram of the flurbiprofen–COX-2 complex reveals that flurbiprofen establishes two dominant hydrogen bonds with residues ARG120 and TYR355, which are maintained approximately 100% and 95.2% of the simulation period, respectively. The stability of the complex is influenced by a single water bridge with residue GLU524 (55% of simulation), as well as by additional hydrophobic interactions established with residues VAL116, VAL349, LEU352, PHE518, and LEU531 (Figure 12A). As discussed before, the molecular docking of chlorogenic acid into the structure of COX-2 is characterized by the formation of three conventional hydrogen bonds with residues ARG120, MET522, and VAL523, as well as by the formation of two additional C-H hydrogen bonds with residues ARG120 and SER530. Among these polar interactions, the highest continuity of 29% is accomplished by ARG120. Moreover, the hydrogen bond established with residue GLU524, otherwise not involved in the molecular docking of chlorogenic acid into COX-2, is maintained for approximately 171.6%. Binding interactions with TYR355 and SER530 are retained with continuities of 59.3% (predominance of water bridge) and 98% (predominance of hydrogen bond), respectively, while the contribution of hydrophobic interactions is insignificant (Figure 12B). On the other hand, the ligand–protein plot of the ellagic acid–COX-2 complex reveals the formation of multiple hydrogen bonds and water bridges with residues ARG120 (30.2% continuity), TYR355 (49.1%), TYR385 (26.9%), TRP387 (37.7%), and SER530 (89.4%), as well as the formation of multiple hydrophobic interactions established with ARG120, VAL349, LEU352, PHE518, and VAL523 (Figure 12C).

The average free binding energy values (∆G_avg_) of the flurbiprofen–COX-1, chlorogenic acid–COX-1, ellagic acid–COX-1, flurbiprofen–COX-2, chlorogenic acid–COX-2, and ellagic acid–COX-2 contacts were computed employing the MM/GBSA methodology and are outlined in Table 10.

## 3. Discussion

Inspired by the traditional use of *Potentilla tormentilla* and its scientifically confirmed anti-inflammatory potential, we aimed to develop a novel delivery system for future applications in inflammation-mediated skin diseases such as psoriasis, atopic dermatitis, eczema, and seborrheic dermatitis [1,4]. Apart from the selection of an efficient natural extract, an important factor that affects treatment outcome is the use of a suitable drug delivery system that will enable sufficient contact between the extract and the skin. In that sense, our extract was incorporated into a gel formulated by a careful selection of polymers that would provide sufficient retention time and excellent spreadability. Poloxamer 407 is a widely used polymer for optimizing drug formulations due to its thermosensitive properties, i.e., the fact that gel based on this polymer exists in a fluid state at low temperatures, while at body or room temperature, it becomes a semi-solid. This behavior is attractive for formulation development, since it ensures the controlled and prolonged release of active substances on the skin [11,12]. The application of poloxamer 407 has certain limitations, such as low mechanical strength and very fast drug release, as well as low durability, while the addition of carbomer leads to improvement of the stability and consistency of the formulation [13]. 

Moreover, combinations of gelling agents such as carbomers and poloxamers already have proven desirable properties: good and easy application, prolonged adhesion, and good permeability. The addition of polyethylene glycol (PEG 400) into the carbomer medium contributes to achieving satisfactory rheological and mucoadhesive properties [14]. The concentration of a combined poloxamer/carbomer gel of 20%/0.15%, respectively, has been proposed for adjusting suitable in situ gelation without runoff upon application [15]. 

Stability evaluation of the formulations is an essential process during product development, since it provides evidence of how the quality of a product varies with time under the influence of specific environmental conditions such as temperature, humidity, and light [16]. In order to assess stability, the developed gel was subjected to determination of organoleptic properties, pH value, conductivity, rheological properties, and centrifugation. The absence of alterations in organoleptic properties indicates that the formulated gel remained stable during the follow-up period. 

Additionally, measuring the pH values of semi-solid preparations for dermal application enables adjusting the optimal pH value for cutaneous products and prevention of skin dehydration, redness, irritability and impairment in bacterial flora induced by skin products with high pH values. Namely, it has been considered that maintenance of an acidic stratum corneum environment promotes ceramide production and adequate hydration and also acts protectively on the skin microbiome. On the other hand, an elevated pH impairs the skin barrier and increases susceptibility to skin infections. Importantly, there are many factors affecting pH values of gel formulations, such as chemical interactions among acidic or alkaline substances present in the formulation, exposure to air leading to oxidation processes, microbial contamination, absorption of carbon dioxide from the air, and temperature changes [10,17,18]. Our designed formulation was confirmed to be safe for dermal application, as pH values measured after storage during three months at different temperatures were in the range of 4 to 6, which meets the optimal requirements for topical products [10].

In order to provide information about the ion transport properties, swelling behaviour, and structural integrity of the gel, we performed electrical conductivity measurements. According to literature data, poloxamer 407 at lower temperatures, below sol–gel transition ones, exists in an aqueous solution, while elevation of the temperature leads to the aggregation of copolymer chains into micellar structures. However, the sol–gel transition temperature (Tsol/gel) varies, and it is affected by the different compositions of poloxamer 407 and other ingredients in the formulation. At room temperature, our formulation existed in a gel state, which is in line with previous data that revealed Tsol/gel was around 24 °C with the poloxamer 407/carbomer ratio of 20/0.15 [15]. On the other hand, during storage at 4 °C ± 2 °C, we detected higher conductivity values, which may be explained by the fact that water can enhance the ionization of certain substances within the formulation, leading to high values of electrical conductivity [19]. Moreover, storing poloxamer 407 on higher temperature leads to dehydration of the hydrophobic polypropylene oxide repeat units and may explain a drop in conductivity over time [12,20]. Changes in conductivity can signal alterations in the network structure, such as crosslinking density or polymer chain conformation, which are important for the mechanical and functional properties of the hydrogel [21]. 

The centrifugation method has been widely used during the process of the evaluation of the stability of pharmaceutical formulations such as gels, since it can be an indicator of different instabilities in the product. In fact, during centrifugation, the test gel sample is subjected to stress that mimics the increased gravity force and particle mobility, which enables detection of potential extract precipitation or possible losses in gel stability, such as decomposition, phase separation, creaming, and sedimentation [16,22]. The absence of alterations in our gel indicates that our gel does not require reformulation and meets the stability requirements. 

The viscosity of our gel decreased with the elevation of shear rate, thus confirming shear thinning or pseudoplastic behavior. This finding suggests a robust network of internal forces established within the samples, ensuring substantial stability. As the shear rate rises, there is a sudden drop in viscosity, a common occurrence in semi-solid formulations. When subjected to shear, the shear stress disrupts the internal network of forces in the sample, causing it to transition from elastic deformation through the yield point into viscous flow. This pseudoplastic rheological behavior is suitable for products intended for dermal application, since it enables the desired application, spreadability, and maintenance on the skin [23]. At shear rates ranging from 100 s^−1^ to 1000 s^−1^, mimicking spreading conditions, viscosities remain low, which facilitates reduced friction and effortless gel application onto the skin. Additionally, viscosities after three months were slightly lower than the initial values, but still similar. This behavior might be attributed directly to the increased entanglement of both polymers, which results in increased polymer resistance to deformation [24]. 

Furthermore, we performed the swelling index measurement because it reflects how a gel interacts with its environment, including its ability to release substances, maintain structural integrity, and deliver the desired properties and stability of gel-based products. The swelling index of a gel can be significantly affected by storage conditions, particularly at different temperatures. We noticed that storage of PGE at 4 ± 2 °C generally reduces the swelling rate of the gel, thus maintaining the stability of the formulation. The gel may swell more slowly and be in a more rigid state due to reduced thermal energy [25]. On the other hand, storing gel samples at 25 ± 2 °C increased swelling, which reflects the porous structure of the gel and may lead to the risk of accelerated degradation or structural changes over time [26].

Performing acute dermal irritation tests allows insight into the potential of cutaneous formulation to induce skin irritation. This study is of great importance, since numerous skin products, especially naturally derived, may cause allergies or a state of skin hypersensitivity that is manifested as edema and erythema [27]. Our findings clearly show that the prepared gel did not lead to either erythema or edema during the 14-day monitoring period. Due to a similarity between humans’ and rats’ responses to cutaneous products, the observed findings in the current study represent valuable data for predicting the likelihood of reactions in human skin [28]. The lack of skin reactions associated with gel application suggests that the gel is not an irritant, which is a significant advantage over available anti-inflammatory products on the market.

Topical application of PEG exerted markedly time-dependent anti-inflammatory potential, as manifested by different percentage inhibitions during the follow-up period. In fact, rat paw edema reduction started from the second hour following carrageenan injection and continued to intensify until the fourth hour. The maximum 39.62% inhibition of paw edema was achieved 4 h after carrageenan injection. Our results are in correlation with previously published research that also confirmed the anti-inflammatory capacity of *Potentilla tormentilla* extract, but in different formulations and experimental models [9,29]. 

According to literature data, the development of the edema induced by carrageenan can be explained by histamine, bradykinin, and cyclooxygenase products up to 2 h following carrageenan administration and by delayed production of arachidonic metabolites between the second and fourth hour. Therefore, the most prominent alleviation of rat paw thickness at the fourth hour can be explained partially through its action primarily by cyclooxygenase 1 and 2 inhibition, as confirmed previously [29,30,31]. Another explanation might involve the initial slower extract release from the formulation due to the mixture of bioadhesive polymers. Tannins, as the dominant compounds in our extract, along with gallic and chlorogenic acids, are likely to significantly contribute to the observed anti-inflammatory capacity of the tested formulation. Recently published data have demonstrated the anti-inflammatory properties of tannins extracted from different plants [32,33]. Specifically, ellagic acid exhibited a pronounced anti-inflammatory effect, ameliorating skin lesions in atopic dermatitis [34] and preventing collagen destruction and inflammation after UV-B radiation [35]. It is assumed that these inhibitory effects of ellagic acid on inflammatory responses were mediated by p38 mitogen-activated protein kinase (MAPK) and signal transducers and activators of transcription (STAT) pathways. In addition, ellagic acid exhibited a potent anti-inflammatory effect against carrageenan-induced inflammation. The mechanisms involved in this protective effect of ellagic acid include the inhibition of leukocyte infiltration and COX-2 expression, as well as the suppression of TNF-α and IL-β production [36].

Preliminary molecular docking results suggest that gallic acid and rutin exhibit significantly lower binding affinity towards COX-1 and COX-2 isozymes compared to flurbiprofen. According to calculated docking scores for chlorogenic acid (−28.45 kJ/mol) and ellagic acid (−26.36 kJ/mol), it is evident that the investigated molecules display a lower binding capacity against COX-1 but a comparable binding affinity towards COX-2 (−34.72 kJ/mol and −34.22 kJ/mol, respectively) in relation to flurbiprofen (−40.58 kJ/mol for COX-1 and −38.07 kJ/mol for COX-2). Based on obtained docking scores, it can be also observed that the examined polyphenols generally exhibit a higher binding affinity for COX-2, with the exception of gallic acid, which accomplishes nearly identical docking scores. Notably, chlorogenic acid and especially rutin, distinguished by their larger molecular sizes relative to gallic acid, exhibit significantly lower docking scores during the molecular docking into COX-2. This result can be explained by the fact that the inhibitor binding site in COX-2 is nearly 20% larger than in COX-1 [37], so rutin cannot accommodate the COX-1 binding site due to its voluminous molecular structure. In an *in silico* investigation by Vyshnevska and colleagues [38], gallic acid demonstrated a nearly equivalent docking score during molecular docking into COX-2 (−26.36 kJ/mol), as observed in our study (−25.94 kJ/mol). In a study with a design similar to ours [39], scientists performed phytochemical profiling and bioactivity exploration of *Rabelera holostea* extract, wherein chlorogenic acid and rutin were modeled into the active sites of COX-1 and COX-2. Namely, chlorogenic acid and rutin accomplished significantly lower docking scores (COX-1: −41.96, COX-2: −45.73 kJ/mol and COX-1: −26.42, COX-2: −17.28 kJ/mol, respectively) compared to our findings (COX-1: −28.45, COX-2: −34.72 and COX-1: −10.88, COX-2: −27.61 kJ/mol). In this study, chlorogenic acid established equivalent hydrogen bonds with residue ARG120 in the active sites of COX-1 and COX-2, consistent with our observations. In accordance with our findings, during molecular docking of rutin into COX-1 and COX-2, relatively high values of docking scores were calculated due to the presence of unfavorable steric bumps. The docking study conducted by El-Shitany and colleagues [36] revealed a high affinity of ellagic acid towards COX-2, consistent with our observations, but with different hydrogen bonds established into the active site of COX-2. Although ellagic acid formed an identical hydrogen bond with residue SER530 as identified by the present *in silico* study, the molecule established additional hydrogen bonds with residues ARG120, TYR355, and TYR385, which were not observed in our binding analysis.

Molecular dynamics data obtained for the flurbiprofen–COX-1 complex indicate that the persistence of four hydrogen interactions of flurbiprofen with the residues ARG120 (triple hydrogen bond) and TYR355 (single hydrogen bond) significantly contributes to the high stability of the formed complex with COX-1. Therefore, the lower stability of the corresponding chlorogenic and ellagic acids complexes, in comparison to the flurbiprofen–COX-1 complex, can be explained by the absence of these permanent hydrogen bonds during the simulation time. On the other hand, observed ligand–protein interactions clearly suggest the formation of a more stable ellagic acid–COX-2 complex in comparison to the corresponding flurbiprofen complex. The enhanced stability of the mentioned complex arises from the presence of permanent hydrogen bonds, water bridges, and multiple hydrophobic interactions. Calculated MM/GBSA energy values confirm the results of preliminary molecular docking data in terms of the binding affinity of flurbiprofen, chlorogenic acid, and ellagic acid towards COX-1. However, MM/GBSA results lead to contrasting conclusions regarding the binding affinity of flurbiprofen, chlorogenic acid, and ellagic acid against COX-2. Specifically, chlorogenic acid and particularly ellagic acid achieve notably lower values of MM/GBSA energy compared to flurbiprofen, suggesting their potential to form more stable complexes with the COX-2 isozyme over time, in comparison to flurbiprofen.

## 4. Materials and Methods

### 4.1. Plant Materials and Extract Preparation

The plant material used in this research, dried rhizome of *Potentilla tormentilla*, was obtained from Institute for Medicinal Plants Research, Dr. Josif Pančić, Belgrade, Serbia. The dried rhizome of *Potentilla tormentilla* was pulverized and the ethanolic extract was prepared using ultrasonic extraction, with 70% aqueous ethanol as a solvent. The dry extract was obtained by evaporation under reduced pressure (RV05 basic IKA, IKA^®^ Werke GmbH& Co., Staufen im Breisgau, Germany) and used for the preparation of the semi-solid formulation.

### 4.2. Chemical Characterization of Potentilla Tormentilla Extract

#### 4.2.1. Preliminary Chemical Characterization–Total Phenolics and Tannins

The total phenolics content (TPC) in the obtained extract was quantified spectrophotometrically (UV–VIS Spectrophotometer 8453 (Agilent Technologies, Santa Clara, CA, USA)), according to the previously described approach based on the Folin–Ciocalteu reagent [40]. The results were expressed as milligrams of gallic acid equivalents per g dry weight (DW), based on the previously constructed calibration curve for gallic acid under the same experimental conditions. Triplicate measurements were performed and results were presented as mean values ± standard deviation (SD).

The content of total tannins (TTC) was determined using the method described in the European Pharmacopoeia [41]. Briefly, decoctions prepared from the investigated extracts were treated with phosphomolybdotungstic reagent in an alkaline medium after and without treatment with hide powder. The absorbance of the obtained solutions was measured at 760 nm, while the percentage content of total tannins, expressed as pyrogallol (%, *w*/*w*), was calculated from the difference in absorbances of the solution containing total polyphenols and the solution containing polyphenols not adsorbed by hide powder. The reported results represent the mean of three determinations ± SD.

#### 4.2.2. HPLC Analysis of *Potentilla tormentilla* Ethanolic Extract

Two liquid chromatography-based (HPLC-DAD) analytical methods were applied in order to perform detailed chemical characterization of the obtained *Potentilla tormentilla* ethanolic extracts.

The first method, as previously described [42], was used for the quantification of phenolic acids (caffeic acid, gallic acid, chlorogenic acid, trans-cinnamic acid, rosmarinic acid, *p*-coumaric acid, ferulic acid), flavonoids (quercetin), and flavonoid glycosides (quercitrin and rutin). Briefly, the extract was analyzed by an Agilent Technologies 1100 device (Agilent Technologies, Santa Clara, CA, USA) using a Nucleosil C18 column (250 mm × 4.6 mm, particle size 5 μm). The mobile phase system consisting of 1% aqueous solution of formic acid (A) and methanol (B) was delivered in gradient mode, whereas the volume of the sample’s injection was 10 µL. The chromatograms were monitored at three specific wavelengths: 280 nm, 330 nm, and 350 nm, while the compounds of interest were quantified based on external standard calibration solutions analyzed under the same experimental conditions. All of the required chemical standard substances were obtained from Sigma Aldrich (Sigma Aldrich, Burlington, MA, USA).

The second method was used for quantification of epicatechin and ellagic acid in the obtained *Potentilla tormentilla* ethanolic extract. The sample was analyzed by a 1200 HPLC system (Agilent Technologies equipped with a LiChrospher^®^ 100, RP-18e (5 µm, 250 × 4) column) using two mobile phases (phase A being 0.1 M solution of phosphoric acid, and phase B being pure acetonitrile) delivered at 1 mL/min, according to the following programme: 89–75% A (0–25 min); 75–60% A (25–30 min); 60–35% A (30–35 min). The volume of injection was 10 µL, while the chromatograms were monitored at 260 nm. The compounds were quantified based on the corresponding external calibration standards analyzed under the same experimental conditions [43].

### 4.3. Formulation of Potentilla tormentilla Extract-Based Gel

**Figure 13:**
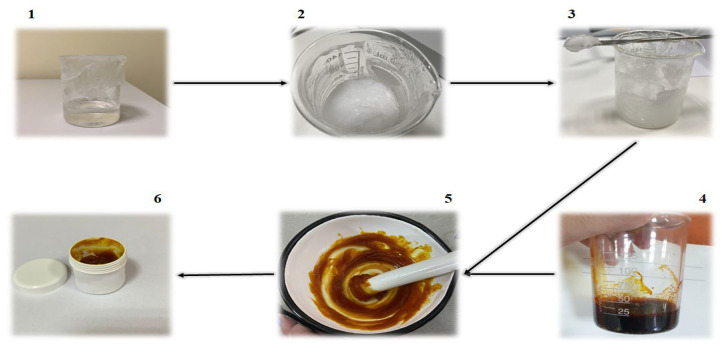
Preparation process steps. 1—carbopol dispersion; 2—addition of poloxamer 407 to carbopol dispersion; 3—prepared gel base; 4—*Potentilla tormentilla* extract; 5—stirring *Potentilla tormentilla* extract with gel base; 6—prepared *Potentilla tormentilla* gel.

The composition of the *Potentilla* extract-based gel (PEG) is presented in Table 11. The PEG was prepared by dispersing carbomer 940 in propylene glycol and purified water. The carbopol dispersion was kept at rest for 24 h to allow for complete swelling [44]. After that, it was slowly dispersed in the carbopol dispersion and allowed to rest at 4 °C for 24 h [45]. The obtained gel was neutralized with required quantity of triethanolamine to obtain pH 5.0 to 5.5. Finally, the *Potentilla tormentilla* extract was added slowly with continuous blending until a homogenous hydrogel was obtained (Figure 13). Mixing carbomer 940, poloxamer 407, and extract was conducted using the IKA RW 20 digital propeller laboratory mixer (IKA^®^-Werke GmbH & Co. KG, Staufen, Germany) with a mixing speed of 400 rpm. 

The carbomer 940 and propylene glycol were purchased from Avena Lab^®^ (Vrsac, Serbia). Poloxamer-407 and triethanolamine were received from Sigma-Aldrich (St. Louis, MO, USA). All reagents were of analytical grade.

### 4.4. Assessment of Stability of PEG Formulation

The developed gel was evaluated for long-term stability that included organoleptic properties such as colour, odour, pH value, conductivity, and reological properties 24 h after preparation and after 90 days of storage in a well-closed plastic box. A sufficient amount of gel was stored in a refrigerator at a temperature of 4 ± 2 °C and 25 ± 2 °C in a thermostatically controlled oven according to guidelines [11,46,47,48]. The accelerated stability of the developed gel was performed by centrifugation test [22]. 

#### 4.4.1. Determination of the pH Values

The pH values of the prepared gel were determined using a digital pH meter (Mettler Toledo, Columbus, OH, USA), which was calibrated before use with the standard buffer solution at pH 4.0, 7.0, and 9.0. Measurements of pH value were carried out in triplicate [45].

#### 4.4.2. Determination of the Electrical Conductivity

The electrical conductivity of the PEG was performed using the conductivity device Eutech CON 700 from Thermo Fisher Scientific (Shanghai, China). Measurements were carried out in triplicate at room temperature with a stabilization time of 2 min [46]. 

#### 4.4.3. Centrifugation Test

To perform the centrifugation test, 10 g of PEG was added in a tapered test tube. The PEG was centrifuged twice at 3000 rpm for 15 min at room temperature. Centrifugation was performed with Hettich Mikro 120 equipment, Kirchlengern, Germany. The PEG was subjected to visual inspection to detect any changes such as sedimentation or phase separation [22].

#### 4.4.4. Rheological Characterization 

Rheological characterization of PEG was performed with an Anton Paar MCR 102e Rheometer (Anton Paar, Graz, Austria) equipped with a Peltier temperature device. Rheological measurements were done at constant temperatures of 25 ± 0.1 °C and 37 ± 0.1 °C in order to understand the material’s behavior at room temperature (storage conditions) and at body temperature (physiological conditions). A plate–plate PP25 measuring system was used with a 1 mm gap. Temperature equilibration was carried out for 3 min prior to starting the measurements. Rheocompass version 1.31 software (Anton Paar, Graz, Austria) was used to control the device and acquire measured data [30].

#### 4.4.5. Swelling Index

One gram of PGE formulation was soaked in 5 mL phosphate buffer (pH 5.5) in a Petri dish and left in a dry place for one hour and three hours before measuring the weight. The content was weighed at three time points, and the degree of swelling was established from the formula. The same process was repeated after 90 days with samples stored at 4 ± 2 °C and 25 ± 2 °C [49].

Swelling Index (SW)% = [Wt − Wo/Wo] × 100

(SW) % = Equilibrium percent swelling

Wt = Weight of swollen gel after time t

Wo = Initial weight of gel

### 4.5. In Vivo Experiments

#### 4.5.1. Ethical Statement

This investigation was conducted at the Center for Preclinical and Functional Investigations of the Faculty of Medical Sciences, University of Kragujevac, Serbia. The study protocol was performed in accordance with the regulations of the Faculty’s ethical committee for the welfare of laboratory animals and principles of good laboratory practice and European Council Directive (86/609/EEC).

#### 4.5.2. Animals

Adult male *Wistar albino* rats (250–250 g) between 8 and 10 weeks old were obtained from the Military Medical Academy, Belgrade, Serbia. The rats were kept in stainless steel cages under controlled temperature (22 ± 2 °C), light cycle: darkness 12:12 h, and relative humidity 55 to 60%. Water and food were available ad libitum.

#### 4.5.3. Acute Dermal Irritation of PEG

Six male *Wistar albino* rats were used for the acute dermal irritation test, as described by the Organization for Economic Cooperation and Development (OECD) guidelines 404. Hair from the backs of the rats was clipped, and the rats were left undisturbed for 24 h. After one day, the animals were divided into two groups, depending of the applied formulation:

- BG—animals treated with the gel base;
- PEG—animals treated with 5% *Potentilla* extract-based gel.

A total of 0.5 g of the test formulation was applied to a small shaved area of the skin, and the rats were individually housed. Observations were recorded with special attention during the first 4 h after administration of the preparation. After that, the rats were observed once a day for 14 days. Behavior, general condition, posture, and reflexes were evaluated [50,51]. Additionally, detection of edema and erythema was made and scored according to the Draize scoring system (Table 12) [52]. The scale was 0–4, where 0 indicating the absence of erythema/edema and 4 indicating severe erythema/edema.

#### 4.5.4. Anti-Inflammatory Effects of PEG

The anti-inflammatory potential of PEG was estimated in a model of acute inflammation caused by carrageenan. Paw edema was induced in a left hind paw of each rat by intraplantar injection of 500 μl of 1% carrageenan [53]. All rats were randomly divided into four groups:

- CTRL group—untreated rats;
- HC—rats treated with hydrocortisone ointment 1%;
- BG group—rats treated with gel base;
- PEG group—rats treated with 5% *Potentilla* extract-based gel.

Examined formulations were administered 60 min before the carrageenan injection in an amount of 0.3 g and gently rubbed 50 times with the index finger [29]. The left paw thickness of the rats was measured with a digital vernier caliper (Aerospace, Beijing, China) before injection of carrageenan and after, at different time intervals (1, 2, 3 and 4). The percentage of inhibition of paw edema was calculated according to the formula [53]:

% Inhibition = 100 × [1 − (Yt/Yc)]

Yt—average increase in paw thickness in the treated group of rats between two measurement moments; 

Yc—average increase in paw thickness in the untreated group of rats between two measurement moments.

### 4.6. Statistical Analyses

Statistical analysis of the obtained data was performed by IBM SPSS 20.0 for Windows. The Kolmogorov–Smirnov and Shapiro–Wilk tests were used to examine the normality of data distribution. Data were expressed as means ± standard deviation (X ± SD), and the differences between groups were analyzed by one-way analysis of variance (ANOVA), followed by the Bonferroni test. The difference was considered statistically significant when the *p*-value was lower than 0.05.

### 4.7. In Silico Analyses

#### 4.7.1. Molecular Docking Studies

The binding efficiency of chlorogenic acid, gallic acid, rutin, and ellagic acid towards COX-1 and COX-2 isozymes was determined through molecular docking analysis using AutoDock Vina 1.1.2 software [54]. The energy optimization of the polyphenols’ conformers was conducted utilizing Chem3D Ultra 7.0 software [55], employing the AM1 semi-empirical method. Crystallographic data for COX-1 (PDB ID: 1EQH [56] and COX-2 (PDB ID: 3PGH) [57] were retrieved from the RCSB Protein Data Bank. BIOVIA Discovery Studio [58] was employed to pre-process the target enzymes by removing unnecessary chains, co-crystallized ligands, water molecules, and cofactors from the original crystallographic structures. Final preparation of target proteins prior to molecular docking was conducted by the addition of polar hydrogens and Kollman partial charges in AutoDockTools graphical user interface [59]. A rigid protein–flexible ligand docking protocol was executed, wherein all docking computations were carried out on chain A of the target proteins. Binding pockets on investigated proteins were defined according to the coordinates of the co-crystallized ligand, flurbiprofen. A search area with a grid point spacing of 0.375 Å was set, enclosing a grid box with dimensions of 40 × 40 × 40 points, allowing ligand conformational flexibility. The grid box center coordinates were specified as follows: 26.413, 33.342, and 198.597 for COX-1 and 25.415, 21.973, and 14.891 for COX-2. To assess the inhibitory potential of the investigated polyphenols against COX-1 and COX-2, the following binding parameters were determined: category, type, total number of non-covalent binding interactions, docking scores (∆G in kJ/mol), inhibition constant (K_i_), and ligand efficiency (LE). The three-dimensional non-covalent binding interactions between amino acids within the enzymes’ binding site and the best-docked poses of the investigated compounds were visualized using Pymol 2.5.5 [60].

The inhibition constant is determined based on the value of docking score (∆G in kJ/mol) using the equation: ∆G = RTlnK_i_, where T denotes the temperature set at 298 K, R signifies the gas constant with a value of 1.9872036 10^−3^ kcal K^−1^mol^−1^, whereas K_i_ represents the inhibition constant.

Ligand efficiency quantifies the binding energy per heavy atom of the ligand bound to the target protein: LE = ∆G/N, where N represents the count of heavy atoms in the ligand molecule.

#### 4.7.2. Molecular Dynamics Studies

The best docked chlorogenic acid–COX-1, ellagic acid–COX-1, chlorogenic acid–000COX-2, and ellagic acid–COX-2 complexes served as starting models for molecular dynamics (MD) simulations. As a comparison, the conformational stability of flurbiprofen in its active site of COX-1 and COX-2 was also assessed. The conformational stability of selected complexes over time was assessed using molecular dynamics (MD) studies in Schrödinger Desmond 2020-4 software [61]. The investigated complexes underwent refinement and optimization employing the OPLS3e force field 5 [62], while their solvation and neutralization were carried out applying TIP3P water model 5 [63] and 0.15 NaCl. MD simulations in durations of 30 ns were initiated under constant pressure conditions (1.01325 bar), utilizing recording intervals of 4.8 ps for trajectory analysis and 1.2 ps for energy monitoring. System equilibration under the NPT ensemble was executed at 300 K using the OPLS3e force field. The Prime Molecular Mechanics/Generalized Born Surface Area (MM/GBSA) method was used to estimate the average free binding energy value (ΔG_avg_) for frames extracted from the 15 ns trajectories of the system. The ΔG_avg_ value was calculated by the Prime module of the Schrödinger tool following the equation: MM/GBSA ΔG_avg_ = G_complex_ − G_protein_ − G_ligand_.

## 5. Conclusions

The findings of our study suggest that the gel based on *Potentilla tormentilla* ethanol extract remained stable for up to three months of storage and has no potential for skin irritation. Efficacy of this formulation in inflammation reduction in rats paves the way for its administration as a potentially anti-inflammatory dermatological product in different inflammation-mediated disorders. In addition, obtained *in silico* results indicate that chlorogenic acid and ellagic acid, as polyphenolic compounds present in tested extracts, exhibit superior inhibitory activity against COX-2 then the standard NSAID flurbiprofen. A developed gel based on *Potentilla tormentilla* ethanol extract could be employed as a promising and safe alternative for the treatment of various inflammatory conditions; however, further studies are required before implementation into clinical practice.

## Figures and Tables

**Table 1:** Concentration of compounds identified in *Potentilla tormentilla* ethanolic extract.

Compound	Concentration (mg/g d.w. Extract)
ellagic acid	14.99 ± 1.2
epicatechin	7.8 ± 0.3
ellagic acid derivatives	23.25 ± 1.11
chlorogenic acid	0.152 ± 0.0042
gallic acid	0.34 ± 0.0031
rutin	0.0015 ± 0.00001

d.w.—dry weight.

**Table 2:** Organoleptic characteristics and physical appearance of PEG at different temperature for 90 days.

Parameters	25 °C ± 2 °C		4 °C ± 2 °C	
	1 Day	90 Days	1 Day	90 Days
Colour	Light brown	Light brown	Brownish	Brownish
Odour	Characteristic odor of the extract	Characteristic odor of the extract	Characteristic odor of the extract	Characteristic odor of the extract
Consistency	Semi-solid	Semi-solid	Semi-solid	Semi-solid
Homogenity	No phase separation	No phase separation	No phase separation	No phase separation

**Table 3:** pH values of PEG at different temperatures for 90 days.

		25 °C ± 2 °C	4 °C ± 2 °C
pH	Day 1	5.81 ± 0.14	5.81 ± 0.14
	90 days	5.96 ± 0.16	5.92 ± 0.19

Data showed as mean ± SD (*n* = 3).

**Table 4:** Electrical conductivity of PEG at different temperatures for 90 days.

		25 °C ± 2 °C	4 °C ± 2 °C
Electrical conductivity	Day 1	52.8 ± 0.56 µS/cm	52.8 ± 0.56 µS/cm
	90 days	43.5 ± 0.59 µS/cm	65.9 ± 0.63 µS/cm * ^#^

Data showed as mean ± SD (*n* = 3); *—statistical significance in relation to day 1; #—statistical significance in relation to value at 25 °C ± 2 °C.

**Table 5:** Viscosity values of PEG measured at different shear rates 24 h after preparation and 90 days after storage at 25 °C and 4 °C. Measurements were performed at 25 °C.

	Viscosity of PEG (Pa × s)		
Shear Rate (1/s)	24 h after Preparation	90 Days after Storage at 25 °C	90 Days after Storage at 4 °C
0.101	7239.6	5429.7	4886.73
10	153.14	104.14	88.51
100	20.40	12.85	10.54
1000	2.66	1.59	1.28

**Table 6:** Viscosity values of PEG measured at different shear rates 24 h after preparation and 90 days after storage at 25 °C and 4 °C. Measurements were performed at 37 °C.

	Viscosity of PEG (Pa × s)		
Shear Rate (1/s)	24 h after Preparation	90 Days after Storage at 25 °C	90 Days after Storage at 4 °C
0.101	7902.4	5926.8	5334.12
10	147.87	100.55	85.46
100	12.54	7.90	6.47
1000	1.61	0.967	0.77

**Table 7:** Swelling index of PGE formulation immediately after preparation and 90 days of storage at 4 ± 2 °C and 25 ± 2 °C.

	1st Day			90 Days		
Samples/Hours	0	1	3	0	1	3
PGE	85 ± 0.15	91 ± 0.12	128 ± 0.10			
		
PGE 4 ± 2 °C				84 ± 0.15	80 ± 0.12	75 ± 0.15
PGE 25 ± 2 °C				82 ± 0.22	97 ± 0.14	115 ± 0.18

The values are means of three replicates ± standard deviation.

**Table 8:** Anti-inflammatory activity of PEG in the carrageenan-induced rat paw edema model.

Rat Paw Thickness (mm) (% of Inhibition)					
Groups	0 h	1 h	2 h	3 h	4 h
CTRL	2.52 ± 0.47	4.36 ± 0.41	6.78 ± 0.52	6.17 ± 0.39	6.03 ± 0.48
HC	2.73 ± 0.43	3.69 ± 0.31 (15.36)	4.21 ± 0.42 (37.90) *	3.98 ± 0.36 (35.49%) *	3.18 ± 0.47 (47.26%) *
BG	3.34 ± 0.35	4.18 ± 0.47 (4.12%)	6.27 ± 0.42 (7.52%)	5.99 ± 0.38 (2.91%)	5.67 ± 0.33 (5.97%)
PEG	2.77 ± 0.38	3.62 ± 0.36 (16.97%)	5.43 ± 0.42 (19.91%) *	4.47 ± 0.52 (27.53%) *	3.64 ± 0.47 (39.62%) *

Results are presented as the mean value ± SD (*n* = 8). *—A statistically significant difference at the level of *p* < 0.05 in relation to the control group. CTRL—control; HC—hydrocortisone; BG—gel base; PEG—*Potentilla* extract-based gel.

**Table 9:** Binding attributes for the best-modeled docking poses of investigated compounds within COX-1 and COX-2 binding sites.

Complex	ΔG (kJ/mol)	K_i_ (µM)	LE * (kJ/mol)
Flurbiprofen-COX-1	−40.58	0.076	−2.25
Chlorogenic acid-COX-1	−28.45	10.20	−1.14
Gallic acid-COX-1	−25.52	33.30	−2.13
Rutin-COX-1	−10.88	12,300	−0.25
Ellagic acid-COX-1	−26.36	23.8	−1.20
Flurbiprofen-COX-2	−38.07	0.209	−2.11
Chlorogenic acid-COX-2	−34.72	0.808	−1.39
Gallic acid-COX-2	−25.94	28.09	−2.16
Rutin-COX-2	−27.61	14.30	−0.64
Ellagic acid-COX-2	−34.22	0.997	−1.55

* LE—ligand efficiency.

**Table 10:** MM/GBSA average binding energies (∆G_avg_) of flurbiprofen–COX-1/2, chlorogenic acid–COX-1/2, and ellagic acid–COX-1/2 contacts.

Protein	Ligand	MM/GBSA ΔG_avg_ ± SD * (kJ/mol)
COX-1	Flurbiprofen	−259.86 ± 10.21
	Chlorogenic acid	−241.55 ± 21.07
	Ellagic acid	−235.88 ± 26.53
COX-2	Flurbiprofen	−183.74 ± 11.72
	Chlorogenic acid	−212.32 ± 19.43
	Ellagic acid	−263.96 ± 12.09

* SD—standard deviation.

**Table 11:** Concentration of all components in *Potentilla* extract-based gel formulation (PEG).

Formulation	Components	Quantity (g)
PEG	*P. tormentilla* extract	5
	Poloxamer 407	19
	Carbomer 940	0.14
	Propylene glycol	9.5
	Triethanolamine	q.s.
	Purified water	ad 100

**Table 12:** Draize dermal irritation scoring system.

Score	Translation
0	No erythema or edema
1	Very inappreciable edema or erythema
2	Small edema with raised skin at the edges of the area
3	Moderate to severe erythema or edema
4	Severe erythema or edema

## Data Availability

The data used to support the findings of this study are available from the corresponding author upon request.

## Supplementary Materials

The following supporting information can be downloaded at: https://www.mdpi.com/article/10.3390/ijms25179389/s1.

## References

[1] Michalak M. (2023). Plant Extracts as Skin Care and Therapeutic Agents. Int. J. Mol. Sci..

[2] Petkovic A., Jakovljevic V., Tomovic M., Jeremic J., Ristic G., Bradic J. (2021). Improving Oxidative Stability of Cosmetic Emulsions with Plant Extracts: Current Status and Potential. J. Cosmet. Sci..

[3] Tomczyk M., Latté K.P. (2009). Potentilla—A review of its phytochemical and pharmacological profile. J. Ethnopharmacol..

[4] Kaltalioglu K., Balabanli B., Coskun-Cevher S. (2020). Phenolic, Antioxidant, Antimicrobial, and *In-vivo* Wound Healing Properties of *Potentilla erecta* L. Root Extract in Diabetic Rats. Iran J. Pharm. Res..

[5] Augustynowicz D., Latté K.P., Tomczyk M. (2021). Recent phytochemical and pharmacological advances in the genus *Potentilla* L. sensu lato—An update covering the period from 2009 to 2020. J. Ethnopharmacol..

[6] Hoffmann J., Casetti F., Bullerkotte U., Haarhaus B., Vagedes J., Schempp C., Wölfle U. (2016). Antiınflammatory effects of agrimoniin-enriched fractions of *Potentilla erecta*. Molecules.

[7] Tampa M., Neagu M., Caruntu C., Constantin C., Georgescu S.R. (2022). Skin Inflammation—A Cornerstone in Dermatological Conditions. J. Pers. Med..

[8] Wölfle U., Hoffmann J., Haarhaus B., Rao Mittapalli V., Schempp C.M. (2017). Anti-inflammatory and vasoconstrictive properties of *Potentilla erecta*—A traditional medicinal plant from the northern hemisphere. J. Ethnopharmacol..

[9] Melzig M.F., Böttger S. (2020). *Tormentillae rhizome*—Review for an Underestimated European Herbal Drug. Planta Med..

[10] Lukić M., Pantelić I., Savić S.D. (2021). Towards Optimal pH of the Skin and Topical Formulations: From the Current State of the Art to Tailored Products. Cosmetics.

[11] Marzouk M.A., Osman D.A., Abd El-Fattah A.I. (2018). Formulation and *in vitro* evaluation of a thermoreversible mucoadhesive nasal gel of itopride hydrochloride. Drug Dev. Ind. Pharm..

[12] Chen Y., Lee J.H., Meng M., Cui N., Dai C.Y., Jia Q., Lee E.S., Jiang H.B. (2021). An Overview on Thermosensitive Oral Gel Based on Poloxamer 407. Materials.

[13] Brambilla E., Locarno S., Gallo S., Orsini F., Pini C., Farronato M., Thomaz D.V., Lenardi C., Piazzoni M., Tartaglia G. (2022). Poloxamer-Based Hydrogel as Drug Delivery System: How Polymeric Excipients Influence the Chemical-Physical Properties. Polymers.

[14] Slavkova M., Tzankov B., Popova T., Voycheva C. (2023). Gel Formulations for Topical Treatment of Skin Cancer: A Review. Gels.

[15] Borghi-Pangoni F.B., Junqueira M.V., Ferreira S.B.d.S., Silva L.L., Rabello B.R., de Castro L.V., Baesso M.L., Diniz A., Caetano W., Bruschi M.L. (2017). Preparation and Characterization of Bioadhesive System Containing Hypericin for Local Photodynamic Therapy. Photodiagnosis Photodyn. Ther..

[16] Xavier-Santos J.B., Passos J.G.R., Gomes J.A.S., Cruz J.V.C., Alves J.S.F., Garcia V.B., da Silva R.M., Lopes N.P., Araujo-Junior R.F., Zucolotto S.M. (2022). Topical gel containing phenolic-rich extract from Ipomoea pes-capre leaf (Convolvulaceae) has anti-inflammatory, wound healing, and antiophidic properties. Biomed. Pharmacother..

[17] Choi E.H., Kang H. (2024). Importance of Stratum Corneum Acidification to Restore Skin Barrier Function in Eczematous Diseases. Ann. Dermatol..

[18] Tarun J., Susan J., Suria J., Susan V.J., Criton S. (2014). Evaluation of pH of Bathing Soaps and Shampoos for Skin and Hair Care. Indian J. Dermatol..

[19] Kulawik-Pióro A., Miastkowska M. (2021). Polymeric Gels and Their Application in the Treatment of Psoriasis Vulgaris: A Review. Int. J. Mol. Sci..

[20] Fakhari A., Corcoran M., Schwarz A. (2017). Thermogelling properties of purified poloxamer 407. Heliyon.

[21] Agarwala S. (2020). Electrically Conducting Hydrogels for Health care: Concept, Fabrication Methods, and Applications. Int. J. Bioprinting.

[22] García-Villegas A., Fernández-Ochoa Á., Rojas-García A., Alañón M.E., Arráez-Román D., Cádiz-Gurrea M.L., Segura-Carretero A. (2023). The Potential of *Mangifera indica* L. Peel Extract to Be Revalued in Cosmetic Applications. Antioxidants.

[23] Ferreira L.M., Sari M.H.M., Azambuja J.H., da Silveira E.F., Cervi V.F., Marchiori M.C.L., Maria-Engler S.S., Wink M.R., Azevedo J.G., Nogueira C.W. (2020). Xanthan Gum-Based Hydrogel Containing Nanocapsules for Cutaneous Diphenyl Diselenide Delivery in Melanoma Therapy. Investig. New Drugs..

[24] Jones D.S., Bruschi M.L., de Freitas O., Gremião M.P., Lara E.H., Andrews G.P. (2009). Rheological, mechanical and mucoadhesive properties of thermoresponsive, bioadhesive binary mixtures composed of poloxamer 407 and carbopol 974P designed as platforms for implantable drug delivery systems for use in the oral cavity. Int. J. Pharm..

[25] Yu Y., Feng R., Yu S., Li J., Wang Y., Song Y., Yang X., Pan W., Li S. (2018). Nanostructured lipid carrier-based pH and temperature dual-responsive hydrogel composed of carboxymethyl chitosan and poloxamer for drug delivery. Int. J. Biol. Macromol..

[26] Thang N.H., Chien T.B., Cuong D.X. (2023). Polymer-Based Hydrogels Applied in Drug Delivery: An Overview. Gels.

[27] Lulekal E., Tesfaye S., Gebrechristos S., Dires K., Zenebe T., Zegeye N., Feleke G., Kassahun A., Shiferaw Y., Mekonnen A. (2019). Phytochemical analysis and evaluation of skin irritation, acute and sub-acute toxicity of *Cymbopogon citratus* essential oil in mice and rabbits. Toxicol. Rep..

[28] Ankomah A.D., Boakye Y.D., Agana T.A., Boamah V.E., Ossei P.P.S., Adu F., Agyare C. (2022). Evaluation of Dermal Toxicity and Wound Healing Activity of *Cnestis ferruginea* Vahl ex DC. Adv. Pharmacol. Pharm. Sci..

[29] Hoffmann J., Wölfle U., Schempp C.M., Casetti F. (2016). Tannins from Potentilla officinalis display antiinflammatory effects in the UV erythema test and on atopic skin. J. Dtsch. Dermatol. Ges..

[30] Korhonen M., Hellen L., Hirvonen J., Yliruusi J. (2005). Determination of optimal combination of surfactants in creams using rheology measurements. Int. J. Pharm..

[31] Calvo M.I. (2006). Anti-inflammatory and analgesic activity of the topical preparation of *Verbena officinalis* L.. J. Ethnopharmacol..

[32] Liu S., Wang K., Lin S., Zhang Z., Cheng M., Hu S., Hu H., Xiang J., Chen F., Li G. (2023). Comparison of the Effects between Tannins Extracted from Different Natural Plants on Growth Performance, Antioxidant Capacity, Immunity, and Intestinal Flora of Broiler Chickens. Antioxidants.

[33] Piazza S., Fumagalli M., Martinelli G., Pozzoli C., Maranta N., Angarano M., Sangiovanni E., Dell’Agli M. (2022). Hydrolyzable Tannins in the Management of Th1, Th2 and Th17 Inflammatory-Related Diseases. Molecules.

[34] Ríos J.L., Giner R.M., Marín M., Recio M.C. (2018). A Pharmacological Update of Ellagic Acid. Planta Med..

[35] Bae J.Y., Choi J.S., Kang S.W., Lee Y.J., Park J., Kang Y.H. (2010). Dietary compound ellagic acid alleviates skin wrinkle and inflammation induced by UV-B irradiation. Exp Dermatol..

[36] El-Shitany N.A., El-Bastawissy E.A., El-desoky K. (2014). Ellagic acid protects against carrageenan-induced acute inflammation through inhibition of nuclear factor kappa B, inducible cyclooxygenase and proinflammatory cytokines and enhancement of interleukin-10 via an antioxidant mechanism. Int. Immunopharmacol..

[37] Faki Y., Er A. (2021). Different Chemical Structures and Physiological/Pathological Roles of Cyclooxygenases. Rambam Maimonides Med. J..

[38] Vyshnevska L., Severina H.I., Prokopenko Y., Shmalko A. (2022). Molecular docking investigation of anti-inflammatory herbal compounds as potential LOX-5 and COX-2 inhibitors. Pharmacia.

[39] Katanić Stanković J.S., Đorović Jovanović J., Mišić D., Gašić U., Nikles S., Marković Z., Bauer R. (2023). UHPLC-MS Phytochemical Profiling and Insight into Bioactivity of Rabelera holostea (Greater Stitchwort) Extract. Molecules.

[40] Velioglu Y.S., Mazza G., Gao L., Oomah B.D. (1998). Antioxidant activity and total phenolics inselectedfruits, vegetables, and grain products. J. Agricul. Food Chem..

[41] (2023). European Pharmacopoeia.

[42] Draginic N., Andjic M., Jeremic J., Zivkovic V., Kocovic A., Tomovic M., Bozin B., Kladar N., Bolevich S., Jakovljevic V. (2022). Anti-inflammatory and Antioxidant Effects of *Melissa officinalis* Extracts: A Comparative Study. Iran J. Pharm. Res..

[43] Žugić A., Đorđević S., Arsić I., Marković G., Živković J., Jovanović S., Tadić V. (2014). Antioxidant activity and phenolic compounds in 10 selected herbs from Vrujci Spa, Serbia. Ind Crops Prod..

[44] Plyduang T., Sermkeaw N. (2022). Development and Evaluation of a Hydrogel containing *Momordica cochinchinensis* Spreng Extract for Topical Applications. Braz. J. Pharm. Sci..

[45] Diaz-Salmeron R., Toussaint B., Huang N., Bourgeois Ducournau E., Alviset G., Goulay Dufaÿ S., Hillaireau H., Dufaÿ Wojcicki A., Boudy V. (2021). Mucoadhesive Poloxamer-Based Hydrogels for the Release of HP-β-CD-Complexed Dexamethasone in the Treatment of Buccal Diseases. Pharmaceutics.

[46] Dantas M.G., Reis S.A., Damasceno C.M., Rolim L.A., Rolim-Neto P.J., Carvalho F.O., Quintans-Junior L.J., Almeida J.R. (2016). Development and Evaluation of Stability of a Gel Formulation Containing the Monoterpene Borneol. Sci. World J..

[47] Kaur M., Kaur G., Kaur H., Sharma S. (2013). Overview on Stability Studies. Int. J. Pharm. Chem. Biol. Sci..

[48] Elyashevich G., Rosova E., Zoolshoev Z., Saprykina N., Kuryndin I. (2023). Reversibility of Swelling, pH Sensitivity, Electroconductivity, and Mechanical Properties of Composites Based on Polyacrylic Acid Hydrogels and Conducting Polymers. J. Compos. Sci..

[49] El-Kased R.F., Amer R.I., Attia D., Elmazar M.M. (2017). Honey-based hydrogel: *In vitro* and comparative *In vivo* evaluation for burn wound healing. Sci. Rep..

[50] Andjić M., Draginić N., Kočović A., Jeremić J., Vučićević K., Jeremić N., Krstonošić V., Božin B., Kladar N., Čapo I. (2022). Immortelle essential oil-based ointment improves wound healing in a diabetic rat model. Biomed. Pharmacother..

[51] Nikolic M., Andjic M., Bradic J., Kocovic A., Tomovic M., Samanovic A.M., Jakovljevic V., Veselinovic M., Capo I., Krstonosic V. (2023). Topical Application of Siberian Pine Essential Oil Formulations Enhance Diabetic Wound Healing. Pharmaceutics.

[52] Draize J. (1959). Appraisal of the Safety of Chemicals in Foods, Drugs and Cosmetics.

[53] Nedeljković N., Nikolić M., Čanović P., Zarić M., Živković Zarić R., Bošković J., Vesović M., Bradić J., Anđić M., Kočović A. (2023). Synthesis, Characterization, and Investigation of Anti-Inflammatory and Cytotoxic Activities of Novel Thiourea Derivatives of Naproxen. Pharmaceutics.

[54] Trott O., Olson A.J. (2010). AutoDock Vina: Improving the speed and accuracy of docking with a new scoring function, efficient optimization, and multithreading. J. Comput. Chem..

[55] ChemOffice Ultra 7.0.1, 2002, CambridgeSoft Corporation. http://www.cambridgesoft.com.

[56] Selinsky B.S., Gupta K., Sharkey C.T., Loll P.J. (2001). Structural analysis of NSAID binding by prostaglandin H2 synthase: Time-dependent and time-independent inhibitors elicit identical enzyme conformations. Biochemistry.

[57] Kurumbail R.G., Stevens A.M., Gierse J.K., McDonald J.J., Stegeman R.A., Pak J.Y., Gildehaus D., Miyashiro J.M., Penning T.D., Seibert K. (1996). Structural basis for selective inhibition of cyclooxygenase-2 by anti-inflammatory agents. Nature.

[58] BIOVIA, Dassault Systèmes (2021). Discovery Studio Visualizer, v21.1.0.20298.

[59] Morris G.M., Ruth H., Lindstrom W., Sanner M.F., Belew R.K., Goodsell D.S., Olson A.J. (2009). AutoDock4 and AutoDockTools4: Automated docking with selective receptor flexibility. J. Comput. Chem..

[60] Schrödinger L., DeLano W. PyMOL. https://www.pymol.org/pymol.

[61] (2020). Schrödinger Release 2020-4: Desmond Molecular Dynamics System.

[62] Harder E., Damm W., Maple J., Wu C., Reboul M., Xiang J.Y., Wang L., Lupyan D., Dahlgren M.K., Knight J.L. (2016). OPLS3: A force field providing broad coverage of drug-like small molecules and proteins. J. Chem. Theory Comput..

[63] Jorgensen W.L., Chandrasekhar J., Madura J.D., Impey R.W., Klein M.L. (1983). Comparison of simple potential functions for simulating liquid water. J. Chem. Phys..

