# Supplementary material for: Potentilla tormentilla Extract Loaded Gel: Formulation, In Vivo and In Silico Evaluation of Anti-Inflammatory Properties
Source: Int J Mol Sci. 2024 Aug 29;25(17):9389. doi: 10.3390/ijms25179389 (PMC11395307; doi:10.3390/ijms25179389)
Supplement: Supplementary file 1 [file ijms-25-09389-s001.zip › ijms-3142719-supplementary.pdf]

International Journal of Molecular Sciences

Supplementary Information associated with the paper

Potentilla tormentilla extract loaded gel: Formulation, *in vivo* and *in silico* evaluation of anti-inflammatory properties

Jovana Bradic [1,2], Anica Petrovic [1,2], Milos Nikolic [1*], Nikola Nedeljkovic [1], Marijana Andjic [1,2], Jovan Baljak [3], Vladimir Jakovljevic [2,4,5], Aleksandar Kocovic [1,2], Vanja Tadic [6], Aleksandra Stojanovic [1,2], Igor Simanic[7,8]

1 Department of Pharmacy, Faculty of Medical Sciences, University of Kragujevac, 34000 Kragujevac, Serbia;

2 Center of Excellence for Redox Balance Research in Cardiovascular and Metabolic Disorders, 34000 Kragujevac, Serbia;

3 Department of Pharmacy, Faculty of Medicine, University of Novi Sad, 21000 Novi Sad, Serbia;

4 Department of Physiology, Faculty of Medical Sciences, University of Kragujevac, 34000 Kragujevac, Serbia;

5 Department of Human Pathology, 1st Moscow State Medical, University IM Sechenov, 119991 Moscow, Russia

6 Institute for Medicinal Plant Research "Dr. Josif Pančić", 11000 Belgrade, Serbia;

7 Specialized Hospital for Rehabilitation and Orthopedic Prosthetics, Sokobanjska 17, 11000 Beograd, Serbia;

8 Department of Physical Medicine and Rehabilitation, Faculty of Medical Sciences, University of Kragujevac, 69 Svetozara Markovica St., 34000 Kragujevac, Serbia;

* Correspondence: milos.nikolic@fmn.kg.ac.rs

Contents

HPLC profile of the analyzed sample 3

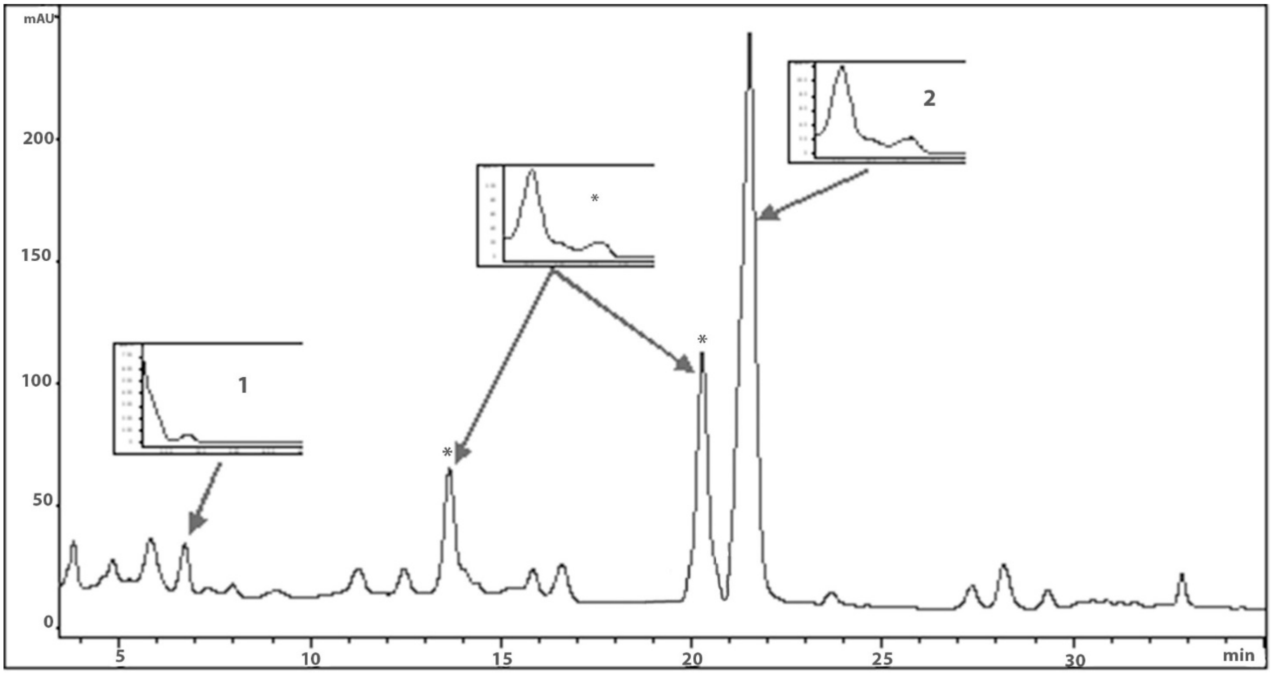

Figure S1. HPLC profile of the analyzed sample. The numbers referred to identified and quantified compounds: 1) Epicatechin; 2) Ellagic acid: *) Ellagic acid derivatives
